# Reactivation of the tRNA^Ser^/tRNA^Tyr^ gene cluster in *Arabidopsis thaliana* root tips

**DOI:** 10.1093/plcell/koaf137

**Published:** 2025-06-06

**Authors:** Guillaume Hummel, Priyanka Kumari, Chenlei Hua, Long Wang, Yan-Xia Mai, Nan Wang, Negjmedin Shala, Emir Can Kaya, Jean Molinier, Jia-Wei Wang, Chang Liu

**Affiliations:** Department of Epigenetics, Institute of Biology, University of Hohenheim, Otto-Sander-Straße 5, Stuttgart, Baden-Württemberg 70599, Germany; Institut de biologie moléculaire des plantes (IBMP-CNRS, UPR 2357), Université de Strasbourg, 12 rue du Général-Zimmer, Strasbourg F-67084, France; Department of Epigenetics, Institute of Biology, University of Hohenheim, Otto-Sander-Straße 5, Stuttgart, Baden-Württemberg 70599, Germany; Department of Plant Biochemistry, Centre for Plant Molecular Biology (ZMBP), University of Tübingen, Auf der Morgenstelle 32, Tübingen D-72076, Germany; National Key Laboratory of Plant Molecular Genetics (NKLPMG), CAS Centre for Excellence in Molecular Plant Sciences (CEMPS), Institute of Plant Physiology and Ecology (SIPPE), Chinese Academy of Sciences (CAS), Shanghai 200032, China; National Key Laboratory of Plant Molecular Genetics (NKLPMG), CAS Centre for Excellence in Molecular Plant Sciences (CEMPS), Institute of Plant Physiology and Ecology (SIPPE), Chinese Academy of Sciences (CAS), Shanghai 200032, China; Department of Epigenetics, Institute of Biology, University of Hohenheim, Otto-Sander-Straße 5, Stuttgart, Baden-Württemberg 70599, Germany; Department of Epigenetics, Institute of Biology, University of Hohenheim, Otto-Sander-Straße 5, Stuttgart, Baden-Württemberg 70599, Germany; Master Study Program “Molecular Biology”, University of Vienna, Universitätsring 1, Vienna 1010, Austria; Department of Epigenetics, Institute of Biology, University of Hohenheim, Otto-Sander-Straße 5, Stuttgart, Baden-Württemberg 70599, Germany; Department of Agricultural Genetic Engineering, Ayhan Şahenk Faculty of Agricultural Sciences and Technologies, Niğde Ömer Halisdemir University, Merkez Campus, Niğde 51240, Turkey; Institut de biologie moléculaire des plantes (IBMP-CNRS, UPR 2357), Université de Strasbourg, 12 rue du Général-Zimmer, Strasbourg F-67084, France; National Key Laboratory of Plant Molecular Genetics (NKLPMG), CAS Centre for Excellence in Molecular Plant Sciences (CEMPS), Institute of Plant Physiology and Ecology (SIPPE), Chinese Academy of Sciences (CAS), Shanghai 200032, China; School of Life Science and Technology, ShanghaiTech University, Shanghai 201210, China; Department of Epigenetics, Institute of Biology, University of Hohenheim, Otto-Sander-Straße 5, Stuttgart, Baden-Württemberg 70599, Germany

## Abstract

Plants maintain redundant tRNA genes (tDNAs) in their nuclear genomes, but the significance, regulation, and functional roles of these genes remain poorly understood. A cluster of tandemly repeated tDNAs decoding serine and tyrosine (SYY cluster) is located on Arabidopsis (Arabidopsis thaliana) chromosome 1, intersecting constitutive heterochromatin and remaining transcriptionally silenced in most tissues. The natural conditions inducing their transcription remain unknown. Here, we elucidate the tissue-specific expression pattern of this cluster during seedling establishment. Our findings reveal that SYY cluster tRNAs are primarily produced in the root cap columella and adjacent root cap cells. Transcriptional reactivation of the SYY cluster occurs in these tissues despite high DNA methylation levels. Furthermore, we demonstrate that these cells accumulate high levels of a transgenic glycoprotein rich in serine, tyrosine, and proline, and that CRISPR/Cas9 deletion of the SYY cluster alters the accumulation and stability of the glycoprotein in these specific cells. Our work provides pioneering evidence of a developmental and cell-specific expression program for a plant tDNA. We offer insights into the putative role of specialized tDNAs in enhancing glycoprotein biosynthesis in protective tissues of the meristem.

## Teaser

Selective activation of transfer RNA genes in root tip cells enables local synthesis of tandem repeat glycoproteins.

IN A NUTSHELL **Background:** Transfer RNAs (tRNAs) are small non-coding RNAs that play a crucial role in protein biosynthesis. When a cell produces a protein, each tRNA carries 1 of the 20 standard amino acids to a “machine” called the ribosome. There, the tRNA pairs with a message carried by messenger RNA (the codon), functioning like pieces of a puzzle coming together. In a cell's DNA, tRNA genes are often present in multiple copies, each located at a different position on the chromosomes.

**Question:** Why do eukaryotes retain so many tRNA genes in their nuclear genome? Are they all functional? If so, how is their usage orchestrated during development and in response to stress?

**Findings:** This study profiles the expression pattern of a group of specific tRNA genes, called “SYY cluster” which is usually inactive in the plant *Arabidopsis thaliana*. These genes are arranged in tandem repeats and are responsible for making 2 types of tRNAs decoding serine and tyrosine amino acids. Normally, the SYY cluster is inactive because its DNA is tightly controlled by methylation. However, we found that in specialized root tip cells, called border-like cells (BLCs), the SYY cluster becomes active. This allows the cells to produce a custom protein containing repeating motifs of serine (S), tyrosine (Y), and proline (P), arranged in an SP_n_Y pattern.

**Next steps:** We aim to uncover the mechanisms that enable the SYY cluster to become active specifically in BLCs. These cells help protect the root tip by making a sticky substance (mucilage) that controls how roots interact with soil microbes. In the future, we want to study how the SYY cluster might support this protective role, possibly by helping make tandem repeat proteins rich in the building blocks serine and tyrosine.

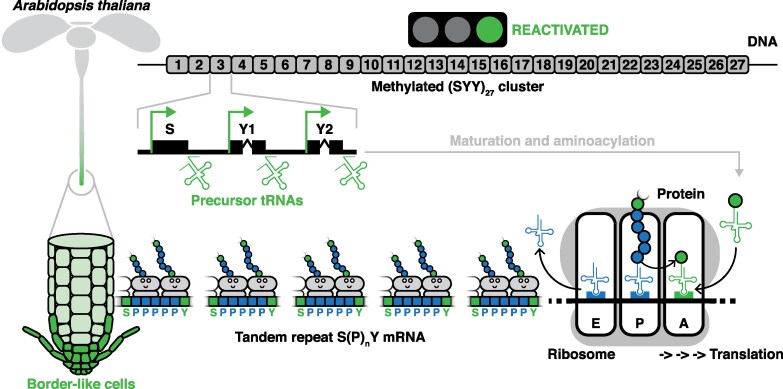

Working model for the regulation and expression of tRNA gene clusters in *Arabidopsis thaliana*. The methylated SYY cluster reactivates in the root tip, with a hotspot in border-like cells. There, serine and tyrosine-decoding tRNAs facilitate ribosomal translation of challenging SPnY motifs, ensuring the expression and stability of tandem repeat glycoproteins.

Bluesky: @guillaumehummel.bsky.social

## Introduction

Transfer RNAs (tRNAs) are ancient non-coding RNAs essential for protein biosynthesis. tRNAs carry a specific amino acid at their 3′ end that is transferred to the C-terminus of nascent polypeptides in ribosomes (Hoagland et al. 1958; Berg et al. 1961; Holley et al. 1965). Each amino acid is decoded by a distinct set of tRNA species (Chan and Lowe 2016).

The evolution of nuclear genomes has led to increased complexity in tRNA gene (tDNA) content, encompassing both sequence diversity and copy number (Goodenbour and Pan 2006; Santos and Del-Bem 2022). tDNAs typically occur in multigenic families, with individual members dispersed across various chromosomal locations (Hummel and Liu 2022; Santos and Del-Bem 2022). Diverse mutational events have expanded the repertoire of unique tRNA sequences among species (Goodenbour and Pan 2006). These tDNAs, which may exhibit polymorphisms, could either preserve tRNA structure or show varying degrees of functional divergence (Chan and Lowe 2016). In the latter case, the resulting “tRNA-like” pseudo-RNAs might exhibit impaired expression, stability, or function in translation.

The copy number of the nuclear tDNAome correlates positively with genome size, protein-coding gene content, and codon frequencies (Goodenbour and Pan 2006; Michaud et al. 2011; Santos and Del-Bem 2022). This suggests that nuclear genomes maintain multiple tDNA copies proportional to the demand for specific amino acids, ensuring sufficient tRNA availability for protein synthesis. However, increasing evidence indicates that the nuclear tDNAome is selectively transcribed in a given species and condition. Chromatin immunoprecipitation of RNA polymerase III-bound complexes and tRNA sequencing show that in humans, approximately half of the 619 tDNAs contribute to less than 1% of total tRNA abundance (Canella et al. 2010; Moqtaderi et al. 2010; Oler et al. 2010; Gogakos et al. 2017; Thornlow et al. 2018; Torres 2019). Similar findings have emerged in plants (Hummel et al. 2020; Liu and Sun 2021; Ma et al. 2021; Hummel and Liu 2022). This prompts the question of why eukaryotes retain such a large number of tDNAs in their nuclear genome. Are some of these genes regulated in a developmental or environmental context? What is the biological significance of individual tDNAs?

The nuclear genome of Arabidopsis (*Arabidopsis thaliana*) Columbia-0 (Col-0) ecotype comprises 5 chromosomes containing 586 tDNAs (Cognat et al. 2022). While most are dispersed along chromosome arms, proline (Pro), serine (Ser), and tyrosine (Tyr) tDNAs form distinct clusters (Green and Weil 1989; Beier et al. 1991; Theologis et al. 2000; Michaud et al. 2011; Hummel et al. 2020; Hummel and Liu 2022). Chromosomes 1 and 2 contain small clusters of proline tDNAs (“P clusters”), and chromosome 1 also has a large, repeated array of serine and tyrosine tDNAs (“SYY cluster,” where “S” and “Y” depict tDNA^Ser^ and tDNA^Tyr^, respectively). These tDNA clusters are located in regions of the genome that epigenetically silence their transcription in seedlings (Hummel et al. 2020). The biological roles of these tDNAs have remained elusive, and it is unknown whether they can be expressed in plants. Here, we investigate the conditions, molecular mechanisms, and physiological significance underlying the SYY cluster's reactivation.

## Results

### The SYY cluster contains a core region that expanded via tandem duplication

**Figure 1.**
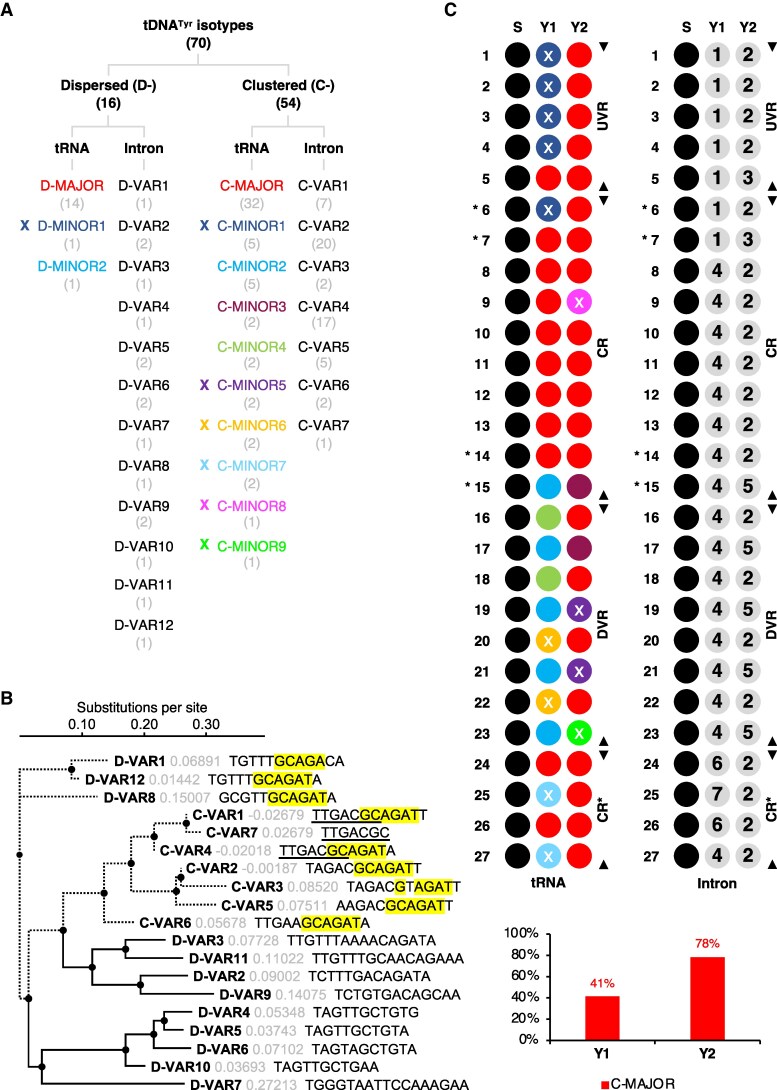
Diversity and organization of tRNA^Tyr^ genes in the Arabidopsis nuclear genome (Col-0 ecotype). **A)** Nuclear tDNA^Tyr^ classification. Numbers in brackets indicate gene copy number for each subcategory. Crosses mark subcategories represented by tRNA-like pseudogenes. **B)** Nuclear tDNA^Tyr^ intron phylogeny. Dotted branches denote shared ancestry between D-VARs 1/8/12 and C-VARs clades. Corresponding sequences share a conserved GCAGAT motif. C-VAR7 originates from truncation of an ancestral intron shared with C-VARs 1 and 4, with the conserved sequence underlined. **C)** Distribution of tDNAs^Tyr^ and introns within the SYY cluster. Colors and numerical annotations follow **A)**. Crosses mark tRNA-like pseudogene locations. Core region (CR), upstream variable region (UVR), downstream variable region (DVR), and CR* are delineated with black arrows. SYY pairs linked to tandem CR expansion (6, 7, 14, and 15) are marked with asterisks. Histograms display the proportion of genes with the C-MAJOR tRNA^Tyr^ sequence at Y1 and Y2 positions. Annotations for all tDNAs^Ser^ and tDNAs^Tyr^ encoded in the *A. thaliana* nuclear genome are available in Supplementary Data Set 1.

Among the 20 tDNA families identified in the Col-0 nuclear genome, tDNA^Tyr^ is the most abundant, comprising 70 genes (Cognat et al. 2022). We classified them based on 3 criteria: genomic distribution (dispersed: D/clustered: C), nucleotide polymorphisms, and copy number (Fig. 1A, Supplementary Fig. S1A). The tDNA with the highest copy number is designated “major,” while others are categorized as “minors.” D-tDNAs^Tyr^ include 1 major (14 copies) and 2 minors (2 copies), whereas C-tDNAs^Tyr^ comprise 1 major (32 copies) and 9 minors (22 copies) (Fig. 1A). Secondary structure analysis showed that 15 of the 16 D-tDNAs^Tyr^ produce functional tRNAs, with D-MINOR1 generating a tRNA-like transcript (Fig. 1A, Supplementary Fig. S1B). In contrast, 13 of the 54 C-tDNAs^Tyr^ (C-MINORs 1, 5 to 9) produce tRNA-like RNAs (Fig. 1A, Supplementary Fig. S1C). We found that D-tDNAs^Tyr^ and C-tDNAs^Tyr^ could also be distinguished with intron variants (VARs). D-tDNAs^Tyr^ exhibit 12 intron VARs, while C-tDNAs^Tyr^ have 7 (Fig. 1, A and B). Notably, a conserved GCAGAT motif is present in all C-tDNA^Tyr^ introns except a truncated 1 in C-VAR7 (Fig. 1B). This motif also appears in D-VARs 8 and 12, which are represented by 1 D-tDNA^Tyr^ each (Fig. 1, A and B). It suggests that the SYY cluster evolved through tandem duplication of 1 or more ancestral tDNAs in Brassicaceae genomes, establishing a phylogenetic link with these 2 D-tDNAs^Tyr^.

The abovementioned tDNA^Tyr^ features allowed us to further reveal the SYY cluster's internal structure (Fig. 1, A and C). We assigned each tDNA^Tyr^ cassette (Y1 and Y2) in individual SYY repeat units a corresponding C-MAJOR/MINOR and an intron C-VAR. We identified a core region (CR) enriched in C-MAJOR pairs, flanked by variable regions with increased C-MINOR and pseudogene content. An adjacent region with additional C-MAJOR pairs was designated CR*. The C-MAJOR is predominantly found at the Y2 position (∼80%), while C-MINORs occur mainly at Y1 (∼60%) (Fig. 1C).

### Root tip cells produce SYY cluster tRNAs during vegetative development

**Figure 2.**
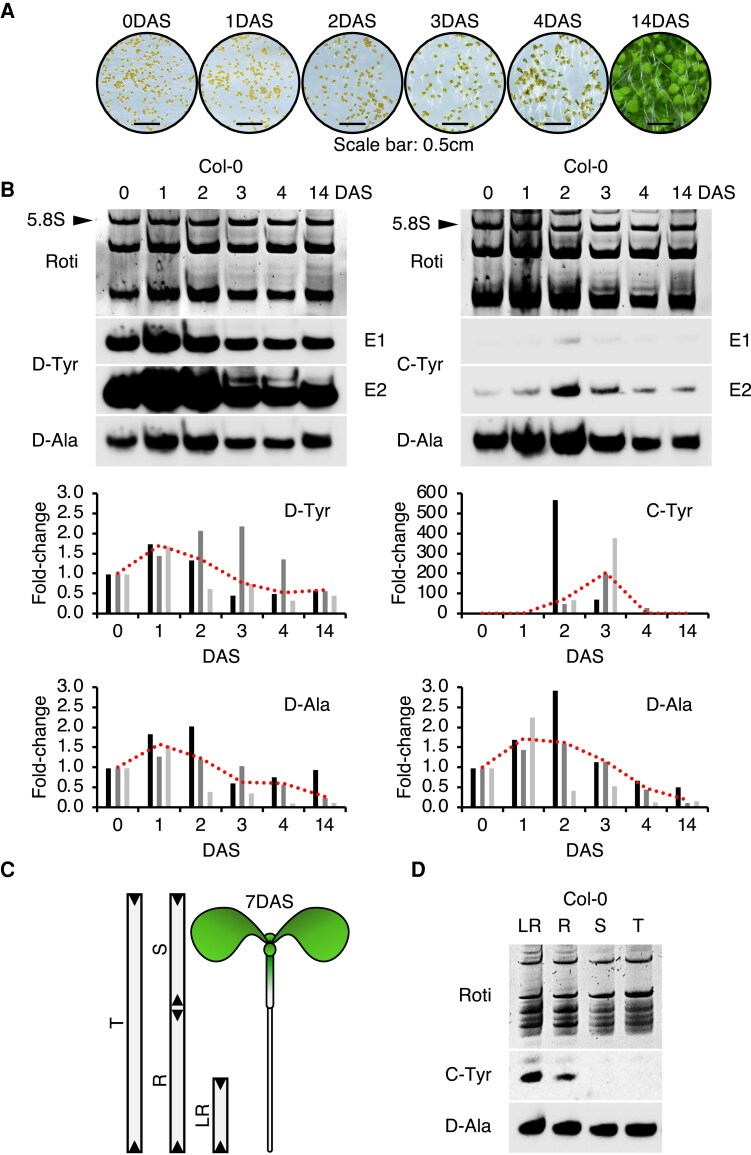
Expression pattern of the SYY cluster during vegetative development. **A)** Representative photos of germination stages at 0, 1, 2, 3, 4, and 14 d after stratification (DAS). **B)** Northern blot analysis of D-tRNAs^Tyr^ (D-Tyr probe), C-tRNAs^Tyr^ (C-Tyr probe), and D-tRNAs^Ala^ (D-Ala probe) in the total RNA from successive germination timepoints. Equal exposures (E1 and E2) are shown for D/C-tRNAs^Tyr^. Results represent 1 of 3 independent experiments. Graphs display quantification of D-Tyr, C-Tyr, and D-Ala signals across 3 biological replicates, normalized to 5.8S rRNA (arrows), and expressed as fold-change relative to 0DAS (histograms). A biological replicate refers to the independent repetition of the experiment under identical conditions. Red dotted lines indicate median values for each timepoint. **C)** Segmentation of a 7DAS seedling. T, total; S, shoot; R, root; LR, lower root. **D)** Northern blot analysis of C-tRNAs^Tyr^ and D-tRNAs^Ala^ in total RNA from specific organs.

The unique sequence variations of D/C-MAJOR tDNAs^Tyr^ enabled us to investigate SYY cluster expression using northern blot and specific probes. In order to decipher the spatio-temporal expression of this SYY cluster, we monitored its expression at 6 timepoints: imbibed seeds (0 d after stratification, DAS), testa rupture (1DAS), endosperm rupture and radicle emergence (2DAS), cotyledon emergence and greening (3DAS), plantlets (4DAS), and adult plants (14DAS) (Fig. 2A).

D-tRNAs^Tyr^ accumulated during germination and remained predominant (Fig. 2B). Interestingly, C-tRNAs^Tyr^ were low but transiently increased at 2 and 3DAS (Fig. 2B). C-tRNAs^Tyr^ peaked with fold-changes of 54 to 572 at 2DAS and 76 to 382 at 3DAS, and were detected in 4DAS only in seed batch #2 (Fig. 2B). In contrast, D-tRNAs^Tyr^ and D-tRNAs^Ala^ displayed similar expression patterns with a slight increase at 1 and 2DAS, followed by a decrease to 14DAS. Their fold changes were modest, ranging from 0.35 to 2.21 for D-tRNAs^Tyr^ and 0.13 to 2.94 for D-tRNAs^Ala^ (Fig. 2B).

To investigate whether the transient accumulation of C-tRNAs^Tyr^ during germination reflects tissue-specific enrichment, we analyzed RNA from whole seedlings (T), shoots (S), whole roots (R), and the lower half of roots (LR) (Fig. 2C). While D-tRNAs^Ala^ accumulated equally across all samples, C-tRNAs^Tyr^ were enriched in R and LR extracts, particularly in the lower part of the root (Fig. 2D). On the other hand, C-tRNAs^Tyr^ were not detectable in T and S RNA extracts. These findings suggest a gradient of SYY cluster expression along the root axis, with C-tRNAs^Tyr^ predominantly accumulated in the lower root regions during the vegetative development of *A. thaliana*.

### The SYY cluster drives C-tRNA^Tyr^ accumulation in roots

**Figure 3.**
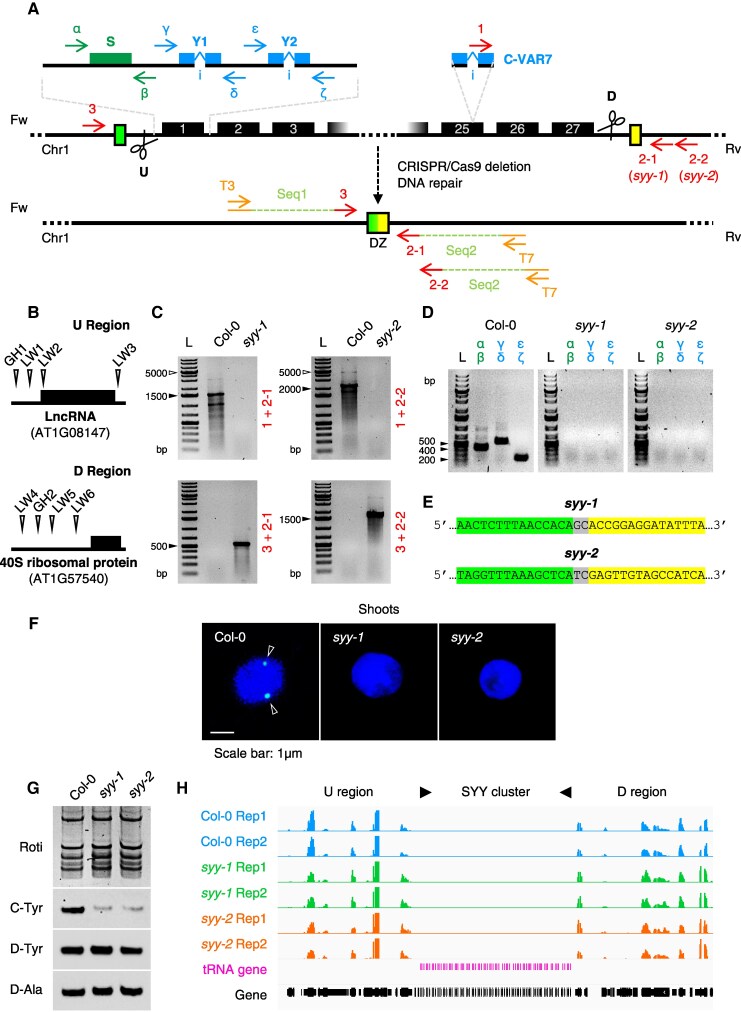
Generation and characterization of tRNA gene deletion alleles. **A)** Screening procedure for lines lacking the SYY cluster. Dual (“GH”) and multiplexed (“LW”) gRNAs targeting U/D regions are shown in **B)** along with nearby genomic features. DZ, deletion zone. **C, D)** PCR profiles of homozygous lines. White arrows indicate putative ladder (L) sizes predicted from the TAIR10 genome. Nevertheless, shorter amplicons were obtained, as indicated by black arrows. Amplicon sizes: 1 + 2−1 = 4543 bp (putative *syy-1*, actual size ≈ 1500 bp); 1 + 2−2 = 5228 bp (putative *syy-2*, actual size ≈ 2000bp); 3 + 2−1 = 547 bp (*syy-1*), 3 + 2−2 = 1875bp (*syy-2*); *α*+*β* = 408 bp; *γ*+*δ* = 564 bp; *ε*+*ζ* = 212 bp. **E)** Sequence of *syy-1* and *syy-2* alleles. DNA junctions (GC/TC dinucleotides) and flanking regions (green/yellow in **A**) are shown. **F)** Subnuclear localization of the SYY cluster in Col-0, *syy-1*, and *syy-2* shoot 2C nuclei. BAC-FISH signals are marked with arrows. Images are representative of ∼100 observations. **G)** D/C-tRNAs^Tyr^ and D-tRNAs^Ala^ levels in Col-0, *syy-1*, and *syy-2* 7DAS roots. **H)** Expression of genes flanking the SYY cluster deletion site (see U/D regions in **A**).

To ascertain that the SYY cluster is the genomic region responsible for the accumulation pattern of C-tRNAs^Tyr^ in roots, knockout mutants bearing the deletion of this region were produced using the CRISPR/Cas9 technology (Fig. 3). Upstream (U) and downstream (D) regions of the SYY cluster were targeted with guide RNAs (gRNAs) (Fig. 3A). Two gRNA strategies were used: dual (1 gRNA per flanking region, GH1 and GH2), and multiplexed (3 gRNAs per region, LW1-LW6) (Fig. 3B) (Zhang et al. 2016; Wu et al. 2018). CRISPR/Cas9 deletions were screened by PCR. Primer pair 1 + 2 would amplify only if the SYY cluster is present with oligonucleotide 1 binding to the truncated C-VAR7 intron, a unique feature of the Col-0 genome (Figs. 1, 3, A and C). Two variants of oligonucleotide 2 were used for each CRISPR strategy: 2-1 for the dual approach and 2-2 for the multiplexed approach (Fig. 3, A and C). Pair 3 + 2 would amplify only if the SYY cluster is deleted (Fig. 3, A and C). We noticed shorter PCR products than expected from wild-type (WT) with the 1 + 2 pair (Fig. 3C), suggesting that the SYY cluster likely lost some repeats in our seed batch. Internal primers α-ζ confirmed the presence/absence of the 3 C-tDNA cassettes (Fig. 3, A and D).

The deletion zone (DZ) was amplified using tailed primers 3 and 2 (Fig. 3A), followed by sequencing, which identified 2 alleles: *syy-1* and *syy-2* (Fig. 3E, Supplementary Fig. S2). *syy-1* (dual gRNAs) excludes the upstream long non-coding RNA but retains the downstream 40S ribosomal protein gene, while *syy-2* (multiplexed gRNAs) preserves both flanking loci. Finally, cytogenetic analysis using fluorescence in situ hybridization (FISH) confirmed SYY cluster deletion. In contrast to WT, the *syy-1* and *syy-2* nuclei did not show FISH signals with probes specifically derived from the SYY cluster (Fig. 3F).

At the RNA level, C-tRNAs^Tyr^ accumulated in WT roots but were depleted in *syy-1* and *syy-2* mutants (Fig. 3G). Nonetheless, a faint residual signal in mutants suggests potential background cross-hybridization. In contrast, D-tRNAs^Tyr^ and D-tRNAs^Ala^ levels are comparable across genotypes (Fig. 3G). Furthermore, RNA-seq experiments verified that SYY cluster deletion did not affect the expression of genes in the vicinity of the deletion site (Fig. 3H).

Altogether, these results demonstrate that the SYY cluster produces C-tRNA^Tyr^ transcripts and underlies the developmental expression pattern observed in Fig. 2. No apparent morphological alterations were observed in these mutants (Supplementary Fig. S3, A to D).

### Border-like cells are hotspots of SYY cluster expression

**Figure 4.**
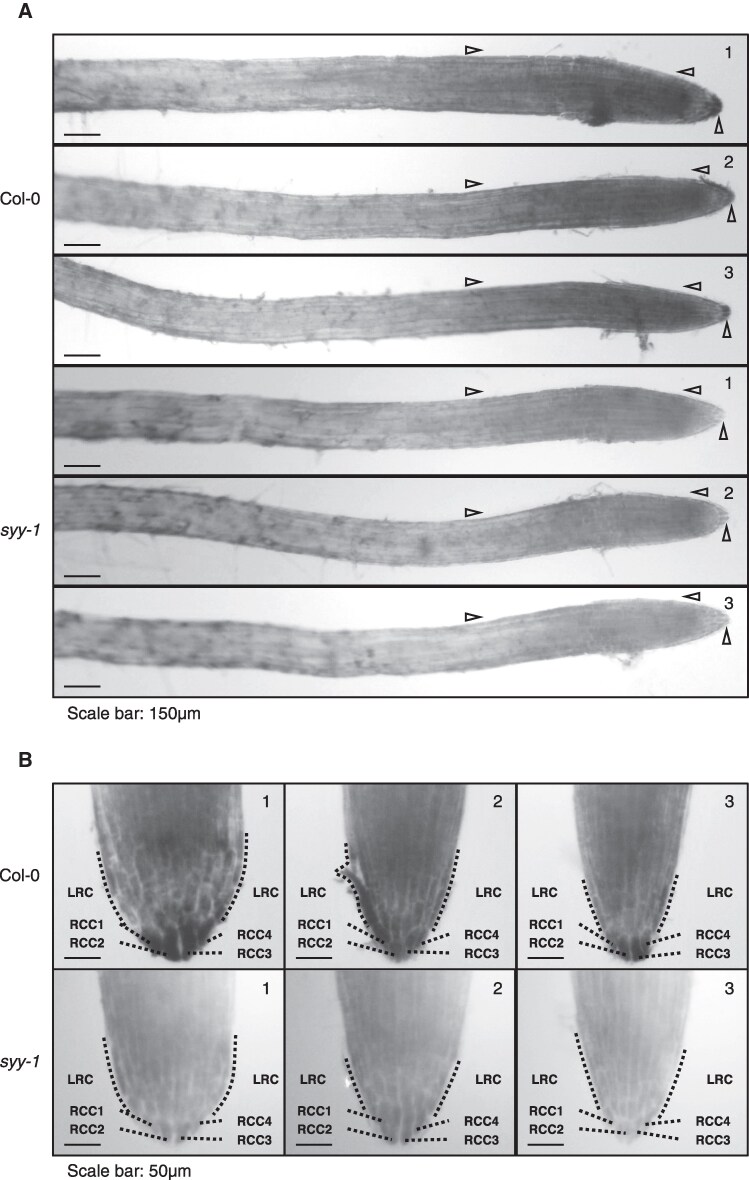
Expression pattern of the SYY cluster in root tissues. **A)** WISH-TSA detection of C-tRNAs^Tyr^ in 4DAS Col-0 and *syy-1* rootlets. Arrows indicate regions of differential staining between genotypes. **B)** Enlarged views of 4DAS Col-0 and syy-1 root tips. Strong C-tRNA^Tyr^ accumulation is highlighted in the 4 outer root cap columella (RCC1-4) cells and lateral root cap (LRC) cells. Three representative images are shown.

To further investigate the spatial expression pattern of C-tRNAs^Tyr^, whole-mount in situ hybridization (WISH) with tyramide signal amplification (TSA) was conducted to examine C-tRNAs^Tyr^ distribution in 4DAS root tissues (Andras et al. 2001; Wójcik et al. 2018). WISH-TSA results revealed a progressive increase in signal intensity along roots (Fig. 4A) consistent with northern blot data (Fig. 2D). Counter-lighting showed homogeneous staining in the division zone and root tip. Notably, the 4 peripheral columella cells (RCC1-4) and adjacent lateral root cap cells (LRC) consistently displayed higher staining than dividing cells (Fig. 4B) (Dolan et al. 1993). In contrast, control *syy-1* roots showed no staining (Fig. 4, A and B). The transcription of the SYY cluster does not appear to play a role in RCC and LRC development and release, as no changes in cellular organization or desquamation pattern were detected in *syy-1* and *syy-2* alleles (Supplementary Fig. S3, E and F).

### Transgenic C-tDNAs^Tyr^ are functional and constitutively active in roots

**Figure 5.**
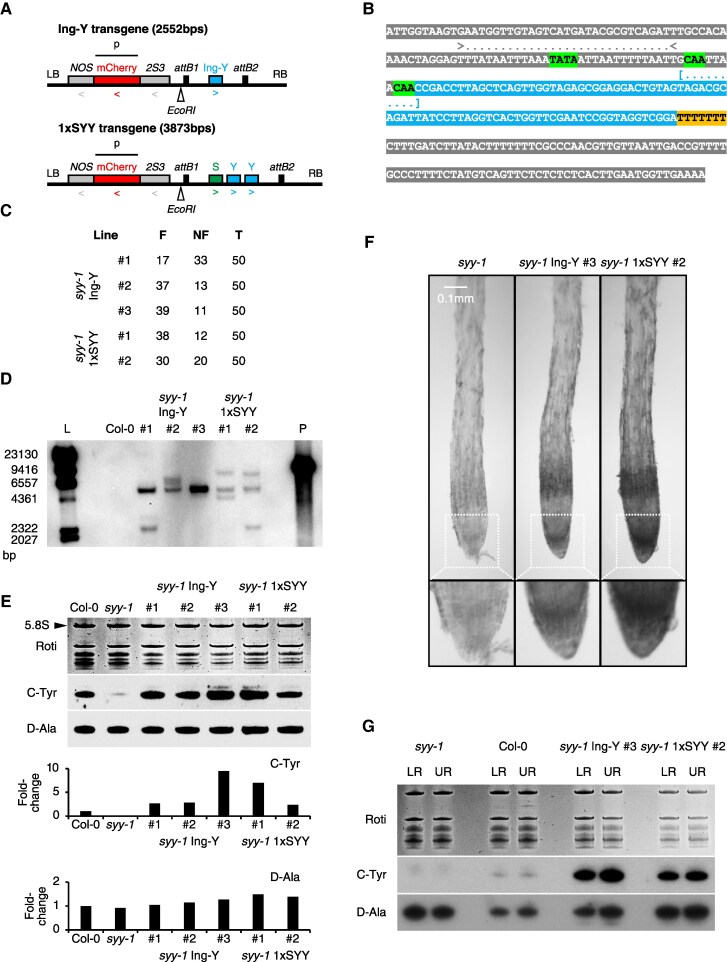
Stable agro-transformation of transgenic tRNA genes. **A)** Genetic map of constructs used for *syy-1* complementation, showing transcriptional orientation (arrows), restriction enzyme site (*Eco*RI), and Southern blotting probe (p). LB, left border; RB, right border; Ing-Y, engineered C-tRNA^Tyr^; *2S3*, *2S albumin gene 3* promoter; *NOS*, *nopaline synthase* terminator. **B)** Sequence of the Ing-Y transgene. Blue sequence represents the major C-tRNA^Tyr^, with intron indicated by brackets. A/T-rich region in the promoter is marked with arrows. TATA-like, CAA, and poly-T motifs are highlighted in green and orange, respectively. **C)** Segregation of non-fluorescent and fluorescent (F) seeds in *syy-1* Ing-Y and *syy-1* 1xSYY lines. **D)** Southern blot analysis of Ing-Y and 1xSYY transgenes in genomic DNA from Col-0 (negative control) and complemented *syy-1* lines. Undigested plasmid (P) used for probe amplification served as the positive control. L, ladder. **E)** Steady-state levels of C-tRNA^Tyr^ and D-tRNA^Ala^ in the roots of 7DAS Col-0, *syy-1*, *syy-1* Ing-Y (3 independent lines), and *syy-1* 1xSYY (2 independent lines). Graphs display quantification of C-Tyr, and D-Ala signals normalized to 5.8S rRNA (arrow), and expressed as fold-change relative to Col-0 (histograms). **F)** WISH detection of C-tRNAs^Tyr^ in 4DAS *syy-1*, *syy-1* Ing-Y, and *syy-1* 1xSYY rootlets (standard method without TSA). **G)** Northern blot detection of C-tRNA^Tyr^ and D-tRNA^Ala^ in lower (LR) and upper (UR) root extracts of 7DAS *syy-1*, Col-0, *syy-1* Ing-Y, and *syy-1* 1xSYY plants.

Next, we asked what expression pattern would C-tDNAs^Tyr^ have if they were not arranged in the SYY tandem repeat format. We either cloned the first repeat unit of the SYY cluster (referred to as “1xSYY”) or engineered a C-tDNA^Tyr^ construct (referred to as “Ing-Y”) (Fig. 5A). The Ing-Y incorporates the C-MAJOR sequence and C-VAR2 intron (dominant in the SYY cluster) combined with the promoter and terminator of a D-tDNA^Tyr^ (AT5G61835) ensuring constitutive plant nuclear tDNA transcription (Figs. 1A and 5B) (Yukawa et al. 2000; Michaud et al. 2011). These constructs were stably introduced into the *syy-1* background, yielding 3 *syy-1* Ing-Y and 2 *syy-1* 1xSYY lines. T2 seed fluorescence suggested single-copy segregation, but Southern blotting revealed multi-insertional lines (Fig. 5, C and D).

Both lines restored C-tRNA^Tyr^ accumulation in root RNA extracts and showed strong levels, while D-tRNA^Ala^ remained unchanged (Fig. 5E). Noticeably, Ing-Y and 1xSYY constructs led to higher C-tRNA^Tyr^ steady-state levels than the endogenous 34-copy repeat (Fig. 5, E to G). This is likely due to their broad and strong expression in the roots of transgenic lines, as observed through standard WISH for spatial patterns (Fig. 5F) and compared with WISH-TSA results (Fig. 4). Northern blot analysis further confirmed high and ubiquitous expression levels (Fig. 5G). Neither *syy-1* Ing-Y nor *syy-1* 1xSYY plants showed C-tRNA^Tyr^ enrichment in LRC and RCC. Instead, they exhibited strong and homogeneous accumulation across root tissues (Fig. 5F). This suggests a developmental regulation mechanism that naturally upregulates SYY cluster expression in these tissues (Fig. 4). Thus, although typically silenced, C-tDNA^Tyr^ loci are functional and capable of strong, ubiquitous expression.

### Natural SYY root expression correlates with high DNA methylation

Previously, we showed that the SYY cluster is subject to DNA methylation (Hummel et al. 2020). To reveal a potential correlation between SYY cluster reactivation and DNA methylation alterations in roots, we performed chop-PCR assays with methylation-sensitive restriction enzymes (MSREs) and reanalyzed bisulfite sequencing data.

**Figure 6.**
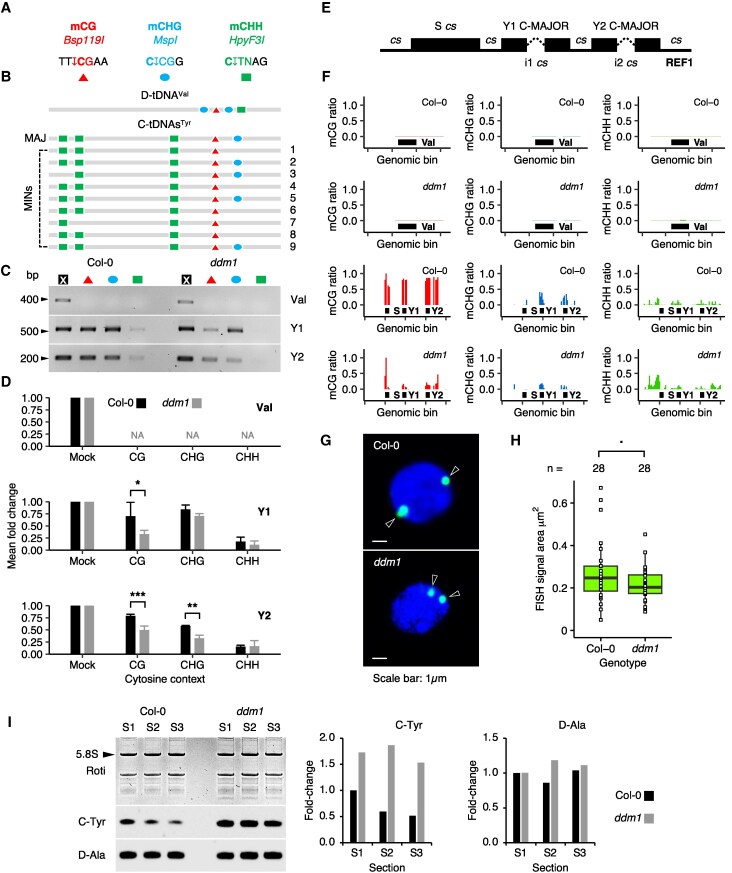
Characterization of SYY cluster chromatin in *ddm1* roots. **A)** Association between methylation context, restriction enzyme, and MSRE site. The methyl-sensitive cytosine is highlighted in bold, and the cutting site is indicated by an arrow. **B)** MSRE site composition of D-tDNA^Val^, major, and minor C-tDNA^Tyr^ sequences. **C)** Chop-PCR amplification of Val, Y1, and Y2 loci from Col-0 and *ddm1* root genomic DNA. Cross indicates mock treatment. Other color and symbol codes are consistent with **A)**. **D)** Relative chop-PCR product quantities by genotype, gene, and cytosine context. Mock is set to 1. Histograms represent the mean of 3 independent biological replicates, with standard deviations shown. NA, not available. A biological replicate refers to the independent repetition of the experiment under identical conditions. A 2-way ANOVA reveals significant differences in Y1 product accumulation upon *Bsp119I* digestion, and Y2 products upon *Bsp119I* and *MspI* digestion between Col-0 and *ddm1* (*P < 0.05; **P < 0.01; ***P < 0.001). Please refer to Supplementary Data Set 2 for a full description of the statistics. **E)** NGS data mapping design for REF1. i: intron, cs: consensus. **F)** Methylation levels at S, Y1, and Y2 loci in Col-0 and *ddm1* root genomic DNA, with D-tDNA^Val^ as the control. Bars represent the ratio of methylated cytosines in 20bps bins. **G)** Subnuclear localization of the SYY cluster in Col-0 and *ddm1* root nuclei. BAC-FISH signals are marked by arrows. Images are representative of ∼100 observations. **H)** Distribution of FISH signal area by genotype. center line: median; box limits: first and third quartiles (Q1, Q3); whiskers: minimum and maximum within 1.5× interquartile range (IQR) from Q1 and Q3; points beyond whiskers: outliers; individual data points: overlaid. Twenty nuclei, each displaying 1 or 2 FISH signals, were analyzed (total acquisitions: *n* = 28). A 1-way ANOVA reveals a significant difference in FISH signal area between Col-0 and *ddm1*, but only for a level of significance ≤ 0.1 (*P* = 0.0843). Please refer to Supplementary Data Set 2 for a full description of the statistics. **I)** Northern blot detection of C-tRNA^Tyr^ and D-tRNA^Ala^ in total RNA extracts from Col-0 and *ddm1* root sections. S1, mitosis + tip; S2, transition zone; S3, elongation zone. Graphs display quantification of C-Tyr, and D-Ala signals normalized to 5.8S rRNA (arrow), and expressed as fold-change relative to Col-0 S1 (histograms).

For chop-PCR, we selected MSRE sites for mCG (*Bsp119I*), mCHG (*MspI*), and mCHH (*HpyF3I*) contexts in C-tDNAs^Tyr^ (Fig. 6, A and B). In parallel, the unmethylated D-tDNA^Val^ (AT3G11395) served as a positive control (Fig. 6B), ensuring complete genomic DNA digestion (Fig. 6, C and D). Our chop-PCR results indicated predominant CG and CHG methylation, but low CHH methylation at Y1 and Y2 C-tDNAs^Tyr^ in WT roots (Fig. 6, C and D). It reflects the typical *A. thaliana* methylation pattern, corroborating prior findings (Law and Jacobsen 2010; Zhang et al. 2018; Hummel et al. 2020). Using *ddm1* templates, chop-PCR detected significant CG and CHG hypomethylation, but no changes in CHH methylation (Fig. 6, C and D).

To verify that DNA methylation dynamics at chosen chop-PCR sites reflect what happens for the entire tDNA cassette, Col-0 and *ddm1* root methylomes were reanalyzed (Zemach et al. 2013). Because the SYY cluster is highly repetitive, TAIR10 reference would undeniably bias the interpretation of DNA methylation levels at genes with identical sequences. To circumvent the issue, we edited a reference REF1 wherein the SYY cluster was replaced by a consensus sequence (Fig. 6E, Supplementary Fig. S4). In REF1, positions with multiple nucleotides in the alignment were considered degenerate (N). Y1 and Y2 tDNA sequences were blocked with the C-MAJOR, while introns i1 and i2 were kept to the consensus but differed by chance. This strategy enabled confident assessment of C-MAJOR methylation status at Y1 and Y2 cassettes. The reanalysis of methylome data confirmed predominant SYY methylation (mCG > mCHG > mCHH), with *ddm1* showing CG and CHG hypomethylation but stable CHH levels (Fig. 6F). FISH signal areas showed minimal statistical differences between Col-0 and *ddm1*, exhibiting high variability, and suggesting that SYY cluster chromatin compaction remains largely unaffected in this mutant (Fig. 6, G and H). This contrasts with the pronounced decompaction observed for this cluster under heat stress (Wang et al. 2023). Similarly, SYY cluster localization near the nuclear periphery appears unchanged in *ddm1*, despite the strong upregulation of C-tDNA^Tyr^ expression across the 3 main root developmental zones: S1 (tip and mitotic zone), S2 (transition zone), and S3 (elongation zone) (Fig. 6I).

**Figure 7.**
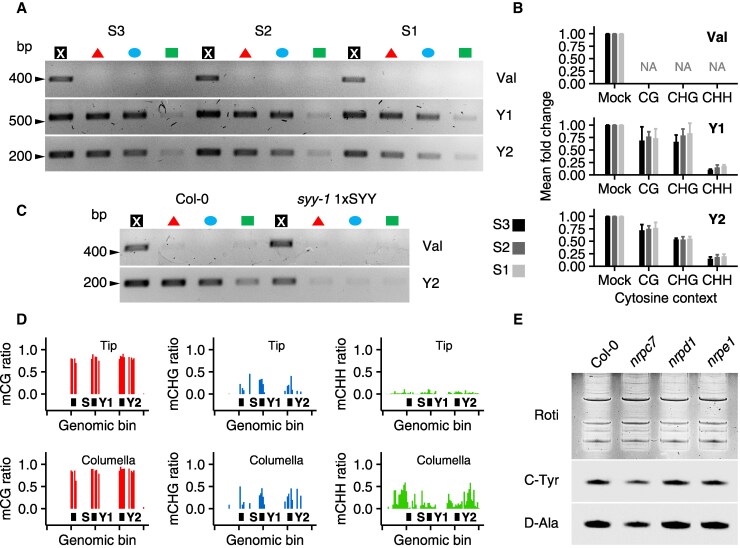
Methylation landscape of natural and transgenic SYY repeats in roots. **A)** Chop-PCR amplification of Val, Y1, and Y2 loci using Col-0 root genomic DNA from S1, S2, and S3 segments. Sample annotation is the same as in Fig. 6C. **B)** Relative chop-PCR product amounts by gene, cytosine context, and root segment. Mock quantification is set to 1. Histograms represent the mean of 3 independent biological replicates, with bars indicating standard deviation. NA, not available. A biological replicate refers to the independent repetition of the experiment under identical conditions. A 2-way ANOVA reveals no significant differences in the accumulation of Y1 and Y2 products, regardless of the root section or enzymatic digestion. Please refer to Supplementary Data Set 2 for a full description of the statistics. **C)** Chop-PCR amplification of Val and Y2 loci using Col-0 and *syy-1* 1xSYY total root genomic DNA. Sample annotation is the same as in Fig. 6C. **D)** Methylation levels at S, Y1, and Y2 loci in whole tip and columella genomic DNA. Bars show the ratio of methylated cytosines in 20bps bins. **E)** Northern blot detection of C-tRNAs^Tyr^ and D-tRNAs^Ala^ in total RNA extracts from Col-0, *nrpc7*, *nrpd1*, and *nrpe1* root S1 region.

Surprisingly, SYY gradient expression in Col-0 roots occurred without significant DNA methylation changes across zones (Fig. 7, A and B), contrasting with strong transgenic 1xSYY expression in the absence of methylation (Figs. 5, F and G, 7C). Furthermore, tissue-specific methylome reanalysis (Kawakatsu et al. 2016) did not show loss of DNA methylation in columella cells, but identified CHH de novo methylation (Fig. 7D).

Collectively, these results underscore the role of DNA methylation in the developmental regulation of the SYY cluster along the root. The analysis of the *ddm1* mutant further establishes the repressive influence of this epigenetic mark on SYY cluster expression. Surprisingly, SYY cluster expression is maintained by a classical RNAPIII transcriptional system, even in the presence of high DNA methylation. This is further illustrated by a partial loss-of-function mutation in a RNAPIII subunit, *nrpc7*, which resulted in a reduction of C-tRNA^Tyr^ and D-tRNA^Ala^ levels in S1 RNA extracts (Fig. 7E). Conversely, knockout mutants for RNAPIV and V subunits (*nrpd1* and *nrpe1*) have no effect on C-tRNA^Tyr^ or D-tRNA^Ala^ accumulation in S1 RNAs, thereby serving as negative controls (Fig. 7E). This comparison highlights the involvement of RNAPIII in the expression of these tRNA species.

### Root tip cells support the translation of Ser-, Tyr-, and Pro-rich proteins

**Figure 8.**
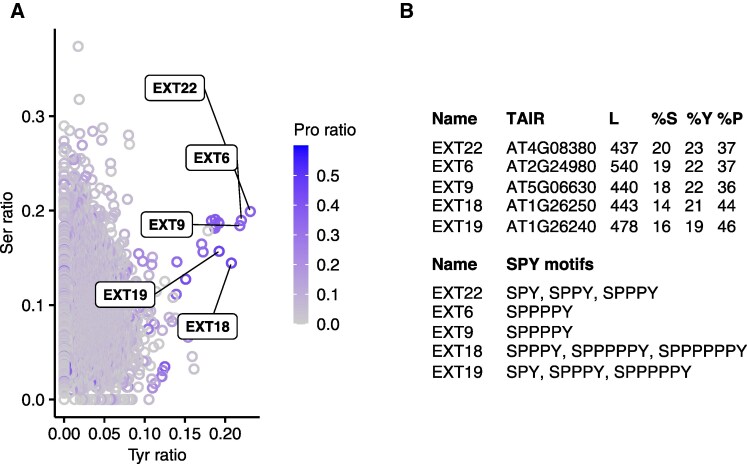
Identification of *A. thaliana* proteins co-enriched with Ser and Tyr amino acids. **A)** Proportion of Ser and Tyr in proteins encoded by the Col-0 genome. A color gradient represents the Pro content of individual proteins, with the top 5 proteins highlighted. **B)** List of EXT names, TAIR IDs, lengths (L), amino acid percentages (%), and SP_n_Y motifs.

The existence of the SYY cluster, which confers the *A. thaliana* genome extra tDNA^Ser^ and tDNA^Tyr^ copies (Hummel et al. 2020), may indicate high translational demands for Ser and Tyr in specific cells or developmental stages. To investigate this hypothesis, we analyzed the *A. thaliana* genome for proteins enriched in these amino acids. A subset of proteins enriched in both Ser and Tyr was identified, with EXTENSINs (EXTs) 22, 6, 9, 18, and 19 as the most prominent (Fig. 8, A and B). EXTs belong to the superfamily of hydroxyproline-rich glycoproteins (HRGPs), which are key components of the cell wall and secreted proteins (Showalter 1993; Showalter et al. 2010; Hijazi et al. 2014; Castilleux et al. 2018; Driouich et al. 2021; Moussu and Ingram 2023). EXTs are characterized by repetitive SP_n_Y motifs that can comprise up to 81% of their sequence (Fig. 8B) (Showalter 1993; Showalter et al. 2010). Notably, the co-enrichment of Pro with Ser and Tyr in EXTs aligns with the presence of tDNA^Pro^ clusters (“P clusters”) in the *A. thaliana* nuclear genome (Fig. 8, A and B).

**Figure 9.**
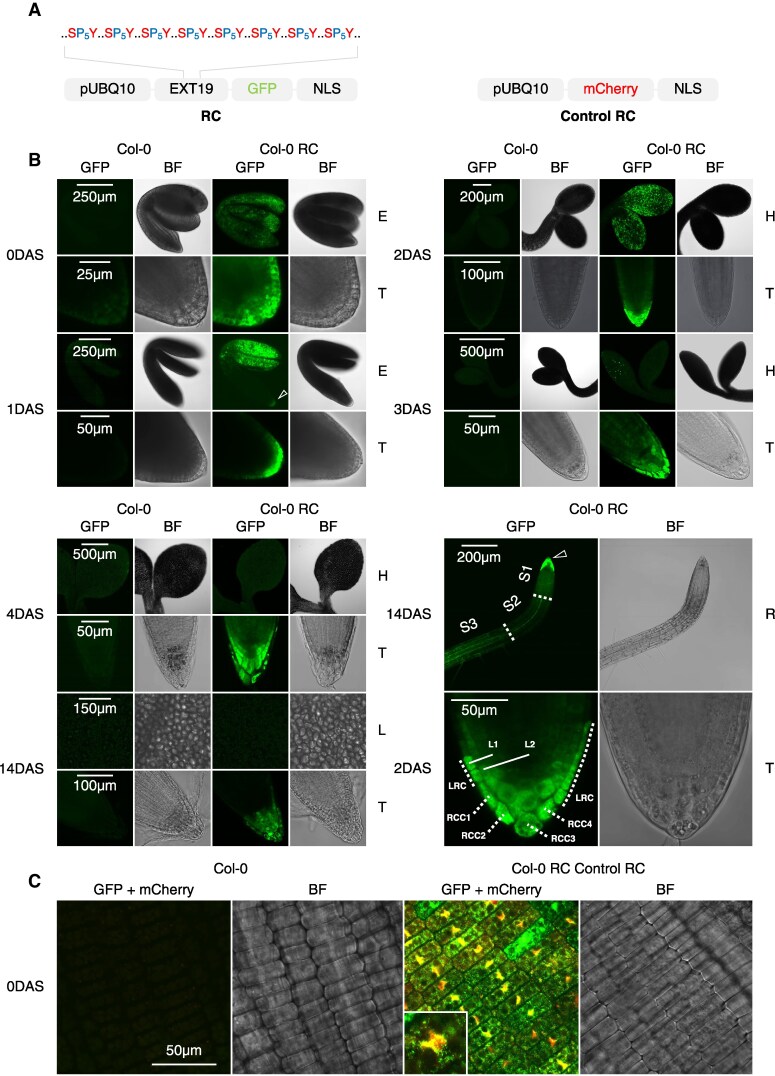
Tissue-specific and subcellular accumulation of reporter proteins. **A)** Simplified reporter constructs (RCs) used to investigate Ser-, Tyr-, and Pro-rich translation in planta (see Supplementary Fig. S5 for full design). **B)** Representative confocal images of Col-0 and Col-0 RC plants at 0, 1, 2, 3, 4, and 14DAS. The white arrow indicates the appearance of RCC and LRC-specific fluorescence. E, embryo; T, tip; R, root; H, hypocotyl; L, leaf. **C)** Representative confocal images of 0DAS Col-0 and Col-0 RC control RC radicle cells, with a magnified view of a single nucleus.

To assess Ser, Tyr, and Pro translation *in planta*, we designed a reporter construct (RC) expressing EXT19 fused to GFP under the constitutive *UBIQUITIN10* promoter (Norris et al. 1993; Sun and Callis 1997) (Supplementary Figs. S5, Fig. 9A). We removed the EXT19 N-terminal secretion signal and added a C-terminal NLS to retain the protein cytoplasmically, preventing its integration into the cell wall. This approach also avoided potential issues with insolubility due to covalent crosslinking of EXTs with HRGPs or other cell wall components (Moussu and Ingram 2023). The fluorescent tag was deliberately placed at the C-terminus of the construct to ensure that its accumulation depends on the prior translation of the Ser-, Tyr-, and Pro-rich region (Supplementary Figs. S5, Fig. 9A). A control RC replaces EXT19-GFP with mCherry, a protein not enriched in Ser, Tyr, or Pro (Supplementary Figs. S5, Fig. 9A).

Stable transformation of Col-0 plants showed strong EXT19-GFP-NLS accumulation in 0DAS embryos, validating ubiquitous expression and translation of RC mRNAs (Fig. 9B). From 1DAS to 14DAS, EXT19-GFP-NLS localized specifically in the peripheral RCC and adjacent LRC cells (Fig. 9B). Expression decreased in cotyledons and was absent in mature leaves and WT plants (Fig. 9B). Despite the NLS, EXT19-GFP accumulated in cytoplasmic foci rather than nuclei, most likely due to competing secretion signals (Fig. 9C).

**Figure 10.**
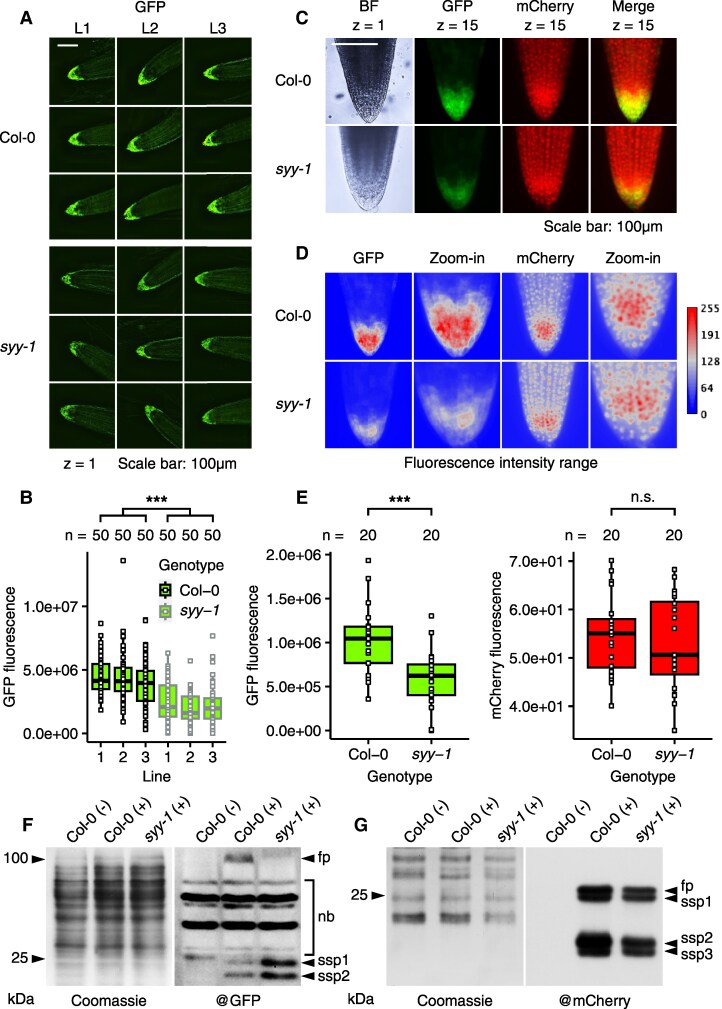
Accumulation of reporter proteins in Col-0 and *syy-1* backgrounds. **A)** Analysis of EXT19-GFP-NLS accumulation in Col-0 and *syy-1* tips by epifluorescence (single focal plane). Three lines (L) are shown per genotype, with 3 representative images for each. **B)** Distribution of GFP fluorescence intensity by genotype and line. Center line: median; box limits: first and third quartiles (Q1, Q3); whiskers: minimum and maximum within 1.5× interquartile range (IQR) from Q1 and Q3; points beyond whiskers: outliers; individual data points: overlaid. Fifty individuals (*n*) per line were analyzed. A 1-way ANOVA reveals a significant difference in GFP fluorescence between Col-0 and *syy-1* (***P < 2 × 10^−16^). Please refer to Supplementary Data Set 2 for a full description of the statistics. Thumbnails in **A)** were selected based on a homogeneous distribution around the medians shown in **B)** (±500,000). **C)** Analysis of EXT19-GFP-NLS and mCherry-NLS co-accumulation in Col-0 and *syy-1* tips by epifluorescence (merged 15 focal planes). BF, bright field. **D)** Range of GFP and mCherry fluorescence intensity in Col-0 and *syy-1* tips. **E)** Distribution of GFP and mCherry fluorescence intensity by genotype. Center line: median; box limits: first and third quartiles (Q1, Q3); whiskers: minimum and maximum within 1.5× interquartile range (IQR) from Q1 and Q3; points beyond whiskers: outliers; individual data points: overlaid. Twenty individuals (*n*) per genotype were analyzed. A 1-way ANOVA reveals a significant difference in GFP fluorescence between Col-0 and *syy-1* (***P = 0.00034) but no significant difference for mCherry fluorescence (n.s.). Please refer to Supplementary Data Set 2 for a full description of the statistics. GFP thumbnails in **C)** were selected based on a homogeneous distribution around the medians shown in **E)** (±150,000). **F)** Immunodetection of EXT19-GFP-NLS in total root extracts from Col-0 and *syy-1* individuals expressing (+) or not expressing (−) the RC. **G)** Immunodetection of mCherry-NLS in total plant extracts from Col-0 and *syy-1* individuals expressing (+) or not expressing (−) the control RC. fp: full protein, nb, nonspecific bands; ssp, subspecies.

Crossing RC lines into *syy-1* resulted in reduced EXT19-GFP-NLS fluorescence in RCC and LRC cells compared with Col-0, while mCherry expression remained unaffected (Fig. 10, A to E). A fluorescence intensity indicator revealed distinct expression patterns: mCherry-NLS accumulated more in the dividing zone at the root tip, while EXT19-GFP-NLS was primarily observed in peripheral RCC and LRC cells (Fig. 10D). This pattern discrepancy confirmed that EXT19-GFP-NLS accumulation is not solely due to the *UBIQUITIN10* promoter activity but linked to the presence of the repetitive Ser/Tyr/Pro-rich sequence. Furthermore, western blot analysis confirmed decreased EXT19-GFP-NLS levels in *syy-1* roots, with additional shorter subspecies suggesting impaired proteostasis and degradation (Fig. 10F). In contrast, mCherry-NLS and related subspecies levels were consistent between Col-0 and *syy-1* (Fig. 10G).

Together, these findings suggest that RCC and LRC cells support the continuous translation of Ser/Tyr/Pro-rich proteins during seedling development. The accumulation of SYY cluster tRNAs would play a critical role by facilitating the translation of repetitive SP_n_Y motifs, ensuring proper EXTENSIN expression and stability in these tissues.

## Discussion

As stated above, we identified that a subset of tRNA^Tyr^ genes, organized in tandem with tRNA^Ser^ genes in a genomic structure termed SYY cluster, exhibits a developmental expression pattern along the roots (Figs. 1 to 6). During seedling establishment, the primary transcriptional hotspots of the SYY cluster are located in the root cap columella and lateral root cap—cell layers bordering protective tissues of the meristem (Dolan et al. 1993). This study reinforces accumulating evidence that the tRNAome is highly dynamic. Only a subset of redundant nuclear tRNA genes is expressed under specific conditions, and this differential contribution is reshaped in response to developmental and environmental cues (Canella et al. 2010; Moqtaderi et al. 2010; Oler et al. 2010; Gogakos et al. 2017; Thornlow et al. 2018; Hummel et al. 2019, 2020; Torres 2019; Torres et al. 2019; Liu and Sun 2021; Ma et al. 2021; Hummel and Liu 2022; Hughes et al. 2023; Kapur et al. 2024).

In this study, we found that the SYY cluster exhibits a gradient expression along the root despite high CG methylation levels (Figs. 6 and 7). This behavior closely resembles that of 5S rRNA gene clusters, which are transcribed by RNAPIII and contain high CG methylation (Mathieu et al. 2002). These findings contrast with an earlier study showing that CG methylation silences clustered tRNA^Tyr^ genes (Hummel et al. 2020). The robust and constitutive expression of the transgenic SYY repeat further supports the inhibitory effect of high DNA methylation on tRNA gene transcription (Figs. 5 and 7). Similar conclusions were reached in *Xenopus laevis* oocytes, where artificially methylated tRNA^Lys^ genes exhibited 80% transcriptional inhibition (Besser et al. 1990). In contrast, methylated 5S rRNA genes remained transcribable both in vivo and in vitro (Besser et al. 1990; Mathieu et al. 2002).

tRNA and 5S rRNA transcription involve distinct RNAPIII promoters and pioneering factors. tRNA genes utilize type-2 promoters, requiring sequential interactions among TFIIIC, TFIIIB, and RNAPIII (Roeder 2019). In contrast, 5S rRNA genes have type-1 promoters, necessitating the additional factor TFIIIA to recruit TFIIIC, TFIIIB, and RNAPIII (Roeder 2019). The transcriptional insensitivity of 5S rRNA genes to DNA methylation might be attributed to a more permissive pre-initiation complex assembly facilitated by TFIIIA binding. However, this hypothesis is challenged by evidence that TFIIIC and TFIIIB can bind methylated type 2-like promoters, and tRNA/Alu-related short interspersed nuclear elements (SINEs) remain expressed despite high levels of DNA methylation in human cells (Varshney et al. 2015). Altering DNA methylation through DNA methyltransferase 1 (Dnmt1) knockout or 5-azacytidine treatment does not activate these elements but instead reactivates silenced protein-coding genes (Varshney et al. 2015). Instead, histone modifications, such as H3K9me3, primarily repress RNAPIII transcription at SINEs (Varshney et al. 2015). Additionally, several TFIIIC-, TFIIIB-, and RNAPIII-bound tRNA genes are located in CpG islands (Sizer et al. 2022), and RNAPIII is recruited to methylated tRNA gene repeats during zygotic genome activation in Xenopus and mouse embryos (Parasyraki et al. 2024).

The SYY cluster is likely not silenced by DNA methylation as even when DNA methylation is disrupted genetically or chemically, tRNA^Tyr^ levels from clustered loci stay low, and P clusters also remain silent (Hummel et al. 2020). While the DNA methylation profile of the SYY cluster remains constant along the root, nucleosome compaction and histone variant composition may undergo dynamic changes during development (Rosa et al. 2014; Probst et al. 2020; Zhang et al. 2021; Feng et al. 2022; Probst 2022; Simon and Probst 2023). Given the subnucleosomal size (∼150 bp) of tRNA genes, their expression may be particularly sensitive to nucleosome positioning and chromatin compaction. Transcribed tRNA genes are typically nucleosome-free (Shukla and Bhargava 2018). In yeast, antisense RNAPII activity at tRNA genes induces histone acetylation, creating a nucleosome-free environment that enhances RNAPIII transcription (Yague-Sanz et al. 2023).

The accumulation of clustered tRNAs^Tyr^ in root cap columella and lateral root cap cells confirms that the chromatin structure in these tissues facilitates the expression of repeats (Fig. 4) (Kawakatsu et al. 2016). In a *ddm1* background, a heterologous DDM1:GFP protein driven by its native promoter accumulates in all root tissues except in the columella and in lateral root cap (Kawakatsu et al. 2016). DDM1 functions as a chromatin remodeler, displacing linker H1 to allow DNA access for methyltransferases such as MET1, CHROMOMETHYLASE 2 and 3, and DOMAINS REARRANGED METHYLTRANSFERASE 2 (Zemach et al. 2013). Additionally, DDM1 facilitates the incorporation of histone variant H2A.W into heterochromatin (Osakabe et al. 2021, 2023). H1 and H2A.W collaboratively promote the compaction of constitutive heterochromatin (Bourguet et al. 2021). The *h1 h2a.w* double mutant exhibits significant heterochromatin decompaction and induces de novo methylation via the RdDM pathway (Bourguet et al. 2021). Notably, columella cells show downregulated mRNA levels of H1.1, H1.2, H2A.W6, and H2A.W7, alongside genome-wide CHH hypermethylation (Kawakatsu et al. 2016). This mirrors the *h1 h2a.w* phenotype, suggesting a globally decondensed nucleosome environment conducive to SYY cluster transcription, despite preserved CG methylation.

Our data demonstrate that root cap columella and lateral root cap cell physiology support the continuous translation and accumulation of a transgenic Ser-, Tyr-, and Pro-rich tandem repeat glycoprotein from the EXTENSIN family (Figs. 8 to 10). We observed a precise correlation between SYY cluster and EXT19-GFP-NLS expression (Figs. 4 and 9). The deletion of the SYY cluster impairs these cells' ability to produce the EXTENSIN (Fig. 10). This deletion mirrors the negative effects of mistranslation, tRNA mismodification, and ribosome quality control defects on the translation of tandem-motif fluorescent reporters in other organisms (fluorescence alterations and proteolytic fragment accumulation) (Choe et al. 2016; Gomes et al. 2016; Miyauchi et al. 2024; Zhang et al. 2024), reinforcing the robustness of our findings. Polyproline motifs in the EXT19 sequence are known to stall ribosomes and require a specialized elongation factor (i.e. EF-P/eIF-5A), likely making their translation sensitive to tRNA availability (Peil et al. 2013; Ude et al. 2013; Starosta et al. 2014; Lassak et al. 2016). Accordingly, tRNA deficiency in *syy-1* may reduce translational efficiency at Ser and Tyr residues flanking polyproline motifs in EXT19, causing ribosome stalling and potential transcript or protein degradation (Vind et al. 2020).

Border-like cells, upon their release from the root cap, produce a mucilaginous network known as the root extracellular trap (Driouich et al. 2011, 2019; Hawes et al. 2016; Castilleux et al. 2021; Hromadová et al. 2021; Fortier et al. 2023). This mucilage, along with the cell wall, forms a key interface for microbial recognition and defense signaling, where hydroxyproline-rich glycoproteins (HRGPs) play a central role. EXTENSINs are upregulated in resistant cultivars upon infection by Potato virus Y or *Colletotrichum sublineolum* (Basavaraju et al. 2009; Otulak-Kozieł et al. 2020), and overexpressing root-specific AtEXT1 in leaves limits *Pseudomonas syringae* DC3000 spread (Wei and Shirsat 2006). Pathogen-induced EXT accumulation leads to oxidative crosslinking at tyrosine residues, forming an insoluble network that strengthens the cell wall and restricts pathogen progression (Mishler-Elmore et al. 2021). Similarly, *P. syringae* DC3000 infection triggers arabinogalactan protein (AGP) upregulation, while Arabinoxylan Pectin Arabinogalactan Protein 1 knockout enhances bacterial growth (Kim et al. 2023).

HRGPs are also secreted in root cap mucilage (Castilleux et al. 2018), contributing to microbial interactions. In potato, HRGP secretion is induced by *Pectobacterium atrosepticum*-derived elicitors (Salam Koroney et al. 2016). AGP accumulation in mucilage may deter pathogens or facilitate symbiosis (Hromadová et al. 2021). Consistently, HRGPs are upregulated in nodules, supporting a role in symbiotic interactions (Castilleux et al. 2018; Hromadová et al. 2021). Notably, nodules in *Medicago truncatula* show genome-wide CHH hypermethylation of repeats, such as transposable elements (Pecrix et al. 2022). A similar pattern is observed in border-like cells, including the SYY cluster (Kawakatsu et al. 2016). This raises the question of whether locus-specific DNA hypermethylation contributes to the epigenomic and transcriptional reprogramming required for plant-microbe interactions in specialized organs and tissues. Future research may investigate the potential involvement of SYY and P tRNA gene clusters in regulating HRGP translation, thus refining the composition and structure of the cell wall and mucilage in response to microbial interactions.

Our study parallels the case of *Bombyx mori* silk glands where specific tRNA^Ala^, tRNA^Gly^, and tRNA^Ser^ species are upregulated, and adapt to the high production of the tandem repeat glycoproteins fibroin and sericin during secretion (Garel et al. 1973; Majima et al. 1975; Siddiqui and Chen 1975; Chevallier and Garel 1982). Similar to the SYY cluster, silk gland-specific tRNA^Ala^ genes are organized in a cluster of tandem repeats (Underwood et al. 1988). This evolutionary convergence between distinct secretory systems highlights a broader principle. The selective amplification and transcriptional mobilization of specialized tRNA gene subsets may represent a conserved strategy to support the synthesis of tandem repeat glycoproteins. Such a mechanism opens additional perspectives on the functionalization of peculiar tRNA gene subsets for the synthesis of tandem repeat glycoproteins (collagen, mucins, etc.) in metazoan secretory systems, with potential applications in synthetic biology, biomaterials, and medical biotechnology.

## Materials and methods

### Plant material and growth conditions

The following Arabidopsis (*Arabidopsis thaliana*) germplasms were used in this study: Col-0 (wild-type), *ddm1-1* (Vongs et al. 1993), *ddm1-2* (Jeddeloh et al. 1999), *ddm1-10* (SALK_093009), *nrpc7-1* (Johnson et al. 2016), *nrpd1-3* (SALK _128428), and *nrpe1-11* (SALK_029919). Seeds were surface-sterilized for 15 min in 70% ethanol and 1% Tween-20, and twice for 2 min in 100% ethanol. They were sown on a half-strength Murashige and Skoog medium pH 5.7 (M0255, Duchefa Biochemie) supplemented with 1% sucrose and 0.6% phytagel (Sigma-Aldrich). Seeds were stratified for 48 h at 4 °C, and vertically grown at 21 °C on a CS 250/300_5_3 (Photon Systems Instruments) cultivation shelf equipped with LEDs (intensity: 100 *µ*mol m^−2^ s^−1^, photoperiod: 16 h).

### Total genomic DNA extraction and sequencing

gDNA was prepared according to (Michiels et al. 2003). Sequencing of vectors, and deletion zone PCR products was performed by Eurofins Genomics.

### Cloning

Dual and multiplexed CRISPR/Cas9 plasmids were obtained as described in (Zhang et al. 2016; Wu et al. 2018). To obtain the Ser, Tyr, and Pro-rich reporter construct, the *UBIQUITIN10* (AT4G05320) promoter and intron-containing 5′UTR, the *EXTENSIN19* (AT1G26240) coding sequence excluding the N-terminal secretion motif and stop codon were amplified from Col-0 gDNA. They were Gibson assembled, together with a GFP-NLS cassette, in a pGREEN-IIS destination vector (Hellens et al. 2000) carrying the Rubisco small subunit (rbcs) terminator, and conferring spectinomycin (bacteria) and Basta (plant) resistances (see pFK-206 in MPI for Biology Tübingen vector list). A double glycine spacer was placed between EXT19 and GFP-NLS genes. The control reporter construct was obtained identically, except that the EXT19-GFP-NLS region was replaced by a mCherry-NLS. The engineered tRNA^Tyr^ locus was produced as gene strands by Eurofins Genomics, and the SYY cluster repeat was amplified from Col-0 gDNA. They were individually cloned into a pFK-206 plasmid by Gibson assembly. A mCherry gene expressed under the control of the seed-specific 2S3 promoter was used as selection marker.

### Southern blotting

The methodology described in (Brown 1993) was essentially applied, with minor adjustments. For each sample, 2.5 *µ*g of gDNA were digested overnight and separated on a 1% agarose gel subsequently treated with depurination and neutralization buffers. Then, gDNA was transferred on a positively charged nylon membrane (Amersham Hybond-N+, GE Healthcare) with a vacuum transfer system (BIO-RAD, Model 785, Vacuum Blotter), and crosslinked with a UVP Crosslinker (AnalytikJena) using following parameters: Energy-1200 (*100*µ*J/cm^2^), twice. A mCherry-specific probe was amplified from a plasmid containing the transgene of interest, and digoxigenin-labeled with DIG High Prime DNA Labeling and Detection Starter Kit II (Roche). Hybridization was performed overnight at 55 °C in hybridization buffer (DIG Easy Hyb, Bottle 7, Roche), followed by stringent washes twice in 2× SSC, 0.1% SDS for 15 min at RT, and twice in 0.5× SSC, 0.1% SDS for 15 min at 55 °C. The membrane was then incubated in blocking buffer (DIG Easy Hyb, Bottle 6, Roche) for 30 min at RT, and further incubated for 30 min at RT in blocking buffer containing Anti-DIG AP conjugate (1:1000, DIG Easy Hyb, Roche). Probes were detected with CSPD substrate (DIG Easy Hyb, Bottle 5, Roche), and with iBrightTM CL750 Imaging System.

### Chop-PCR

For each reaction, 10 ng of gDNA were digested for 30 min at 37 °C in a 10 *µ*L mix containing 0.2 *µ*L of either Fast Digest (FD) *Bsp119I*, *MspI*, *HpyF3I* (Thermo Fisher Scientific), and 1 *µ*L of appropriate buffer. Reactions were inactivated for 20 min at 80 °C, and digested gDNA was isolated with 5 V of SPRI beads (Beckman Coulter). After a 10 min incubation, beads were sedimented with a magnetic stand, and supernatants removed. Beads were cleaned twice in 80% ethanol and air-dried. Elution was done in 10 *µ*L of 10 nm Tris pH 8.0. For each PCR amplification, 1.5 *µ*L were used as template with the ALLin HiFi DNA Polymerase kit (HighQu). Then, 2 *µ*L of 6× DNA loading dye (Thermo Fisher Scientific) were added, and samples were run along with a GeneRuler 1 kb plus DNA ladder (Thermo Fisher Scientific) in a 1% agarose gel containing 0.05 *µ*L/mL ROTI GelStain (Carl Roth). Amplification products were analyzed with a Vilber QUANTUM CX5.

### Isolation of nuclei and fluorescent in situ hybridization

Total nuclei were extracted and sorted using a S3e Cell Sorter (Bio-Rad) as delineated in (Zhu et al. 2017; Wang et al. 2021). FISH experiments were performed with 2C nuclei, and the BAC F9K23 (GenBank: AC082643) as explained in (Montgomery et al. 2022).

### Total RNA extraction

For germination time courses, a protocol yielding high-quality total RNAs (Chang et al. 1993) was used. For other experiments, RNAs were directly TRIzol-chloroform extracted. Concentration and quality were determined with a DeNovix DS-11 FX+.

### RNA sequencing and data processing

Total RNAs were extracted from 7DAS seedlings using the RNeasy Plant Mini Kit (Qiagen). Following DNaseI treatment (Thermo Fisher Scientific, Waltham, MA, USA), total RNAs were incubated with first-strand buffer and subjected to thermal cycling at 80 °C for 2 min, followed by 94 °C for 1.5 min. Polyadenylated RNAs were then isolated from total RNAs and processed according to manufacturer's instructions (NEBNext Ultra II RNA Library Prep Kit for Illumina). After end-repair of double-stranded cDNAs, free ends were ligated to reverse adapters, followed by final enrichment via PCR to construct sequencing libraries. They were sequenced by Novogene.

### Northern blotting

RNAs were routinely loaded in a gel consisting of 15% acrylamide/bis-acrylamide (19/1), 7 m urea and 1× TBE, and resolved by electrophoresis at 150 V constant in 1× TBE. RNA integrity was checked with ROTI GelStain. Then, RNAs were electrotransferred onto a Hybond-N + nylon membrane (Amersham) for 75 min at 300 mA constant and 4 °C in 0.5× TBE. RNA crosslinking was performed with a UVLink 1000 Crosslinker (AnalytikJena, energy mode, 2 × 120 mJ/cm^2^). Probes were hybridized overnight at 55 °C in 6× SSC, 0.5% SDS, and at a final concentration of 20 nm. Washings were performed twice in 2× SSC for 10 min at 55 °C, once in 2× SSC, 0.1% SDS for 30 min at 55 °C, and for 10 min at 25 °C. Then, the membrane was rotated for 1 h at 25 °C in 2× SSC, 0.1% SDS supplemented with 1:15,000 (v/v) HRP-Streptavidin (Abcam), and washed 3 times in 2× SSC, 0.1% SDS for 10 min at 25 °C. Probes were detected with sensitive HRP substrates (Takara), and an iBrightTM CL750 Imaging System. Radioactive northern blot analyses were conducted as previously described (Hummel et al. 2020).

### Whole-mount in situ hybridization coupled to tyramide signal amplification

The WISH procedure was essentially executed as described in (Wójcik et al. 2018), with adjustments. The material was fixed for 3 h in 3.7% formaldehyde, and permeabilized for 90 min at 37 °C in 75 *µ*g/mL proteinase K. The material was hybridized for 16 h at 37 °C with 100 nm biotinylated probe, and washed 3 times for 10 min at 37 °C in 2× SSC, then twice for 30 min at 42 °C in 2× SSC, 0.1% SDS, and 3 times for 10 min in 1× PBS. For the TSA procedure, endogenous peroxidases were first quenched by incubating the material for 1 h in a peroxidase suppressor solution (Thermo Fisher Scientific). After 6 baths of 5 min in 1× PBS, and 1 of 5 min in 1× PBS, 0.1% Tween 20 (PBST) at 25 °C, samples were incubated for 1 h at 25 °C in 1× PBST, 1:15,000 (v/v) HRP-Streptavidin. Upon 6 baths of 5 min in 1× PBST, samples were incubated for 1 h in 1:50 of a digoxigenin-tyramide signal amplification solution (TSA Plus DIG, PerkinElmer) prepared according to manufacturer instructions. After being washed 6 times for 5 min in 1× PBST, and once for 30 min in 1× PBST, 5% BSA, samples were incubated overnight at 4 °C in 1× PBST, 5% BSA, 1:2,500 anti-digoxigenin antibodies fused to the alkaline phosphatase (Roche). Washings were performed twice for 30 min in 1× PBST, 5% BSA and 3 times for 10 min in 1× PBST. Samples were then incubated 3 times for 5 min with 1× TNM buffer (100 mm Tris pH 9.5, 100 mm NaCl, 50 mm MgCl_2_), and staining was performed in 1× TNM supplied with 1:50 NBT/BCIP (Roche). The reaction was finally quenched with 3 baths of 5 min in 1× TE buffer. For the detection of C-tRNAs^Tyr^ in complemented *syy-1* Ing-Y and *syy-1* 1xSYY lines, a regular WISH procedure was executed as described above with a digoxigenin-labeled probe.

### Western blotting

Proteins were extracted using TRIzol-chloroform and resuspended in a buffer containing 4.4 m urea, 11% glycerol, 88 mm Tris–HCl (pH 6.8), 2.2% SDS, and 10 mm DTT. They were stacked in a 6% acrylamide/bis-acrylamide gel (37.5:1) in 125 mm Tris–HCl (pH 6.8) with 0.1% SDS and resolved in a 12% acrylamide/bis-acrylamide gel in 380 mm Tris-HCl (pH 8.8) with 0.1% SDS at 30 mA constant current in a buffer containing 25 mm Tris, 200 mm glycine, and 0.1% SDS. Proteins were then transferred to a methanol-activated PVDF membrane (Immobilon-P) at 4 °C and 80 mA for 45 min. Membranes were stained with 0.1% Coomassie blue in 7% acetic acid/50% methanol, destained in the same solution, and washed in pure methanol. Blocking was done overnight at 4 °C in TBST (TBS + 0.2% Tween) with 5% milk. For GFP detection, membranes were incubated for 1 h with anti-GFP antibody (1:5000; Abcam, ab290), washed 3 times in TBST with 5% milk, incubated for 30 min with anti-rabbit IgG-peroxidase antibody (1:10,000; Sigma-Aldrich), and washed 3 more times in TBST (with and without milk). Detection used HRP substrates (Takara) and the iBright CL750 Imaging System. For mCherry detection, proteins were resolved in a 10% SDS-PAGE gel and transferred to an Immobilon-P membrane. Membranes were incubated with anti-RFP antibody (1:5000; ChromoTek) and anti-mouse IgG-peroxidase antibody (1:10,000; Invitrogen). Signals were detected using Super RX medical X-ray films (Fuji-film).

### Bioinformatics

tRNA sequences, annotations, and intron informations were retrieved from the PlantRNA 2.0 database (Cognat et al. 2022) and TAIR10. Comprehensive annotations for nuclear tRNA^Ser^ and tRNA^Tyr^ loci are available in Supplementary Data Set 1. tRNA structures were predicted using R2DT (Sweeney et al. 2021). Bisulfite datasets were analyzed as described in (Zhu et al. 2017; Hu et al. 2019), except that the reads mapping was conducted with a modified TAIR10 genome reference wherein the SYY cluster was replaced by 1 consensus SYY repeat sequence (Supplementary Fig. S4). Bisulfite files of roots: SRR578936, SRR578937, SRR578926, and SRR578927. Bisulfite files of root tissues: SRR3311822, SRR3311825. RNA-seq reads were aligned against the TAIR10 annotations using TopHat 2 (v2.1.1) with default parameters. The track files of individual RNA-seq samples in bigWig format and the count table were generated with the bam-Coverage function from deepTools and the R package GenomicAlignments, respectively (Kim et al. 2013; Lawrence et al. 2013; Ramírez et al. 2016). ANOVA was performed using the aov function from the default stats package to assess overall differences among group means, followed by the TukeyHSD function to conduct post-hoc pairwise comparisons adjusting for multiple testing (R version 4.3.1, default parameters). Detailed results are reported in Supplementary Data Set 2.

### Phylogenetic analysis

The phylogenetic tree was generated using MAFFT for multiple sequence alignment via the EMBL-EBI portal (Li et al. 2015) with following default settings. Output format: Pearson/FASTA, gap open penalty: 1.53, gap extension penalty: 0.123, order: aligned, tree rebuilding number: 2, guide tree output: on, max iterate: 2, perform FFTS: none. The tDNA^Tyr^ intron alignment is shown in Supplementary File 1, and the script for tree reproduction is provided in Supplementary File 2.

### Microscopy and image analysis

Root tip cell organization was prospected in 10 μg/mL propidium ioide (PI), and images were acquired with an Olympus IX83 microscope supplied with an Olympus XM10 camera and the CellSens Dimension v1.11 software (Olympus). Tip cell release was analyzed in 5% Nigrosin (Sigma-Aldrich), and images acquired with a KERN OPTICS OZL 464 stereo microscope supplied with a tablet Cam ODC 241 (KERN). EXT19-GFP-NLS accumulation pattern was captured using an LSM880 confocal microscope (Zeiss) and ZEN black v2.3 software. Detailed confocal microscopy settings are provided in Supplementary Data Set 3. FISH results were captured with an LSM700 confocal microscope. EXT19-GFP-NLS and mCherry-NLS accumulation in Col-0 and *syy-1* backgrounds was analyzed similarly to the PI experiments, or with an LSM780 confocal microscope (Zeiss). Detailed confocal microscopy settings are provided in Supplementary Data Set 3. WISH images were taken like the Nigrosin experiments. To quantify root tip GFP and mCherry fluorescence, images were first converted in 8bits, and the “threshold” function of ImageJ was used with following parameters: default, B&W, and dark background. Integrated density was then measured in a standardized area. The area of FISH signals was measured by manually outlining them using ImageJ. Finally, blot quantifications were performed using the densitometric analysis tool of ImageJ, using unsaturated exposures whenever possible.

### Miscellaneous

Information related to oligos used in this work can be found in Supplementary Data Set 4. They were either synthetized by Eurofins or Sigma-Aldrich.

### Accession numbers

Source data related to this study are available under project PRJNA1166896. The count table is available in Supplementary Data Set 5.

## Supplementary Material

koaf137_Supplementary_Data

## Data Availability

The data underlying this article will be shared on reasonable request to the corresponding authors.

## Supplementary data

The following materials are available in the online version of this article.

**Supplementary Figure S1.** Sequence and secondary structure of putative mature nuclear tRNA^Tyr^ transcripts.

**Supplementary Figure S2.** Raw deletion zone sequences.

**Supplementary Figure S3.** Phenotyping of tRNA gene mutants during development.

**Supplementary Figure S4.** Consensus sequence used for the mapping of NGS data.

**Supplementary Figure S5.** Design of a reporter system to monitor Ser, Tyr, and Pro-rich translation.

**Supplementary Figure S6.** Uncropped blots.

**Supplementary Data Set 1.** Annotation of dispersed and clustered serine (S) and tyrosine (Y) tRNA genes in the nuclear genome of A. *thaliana* accession Col-0.

**Supplementary Data Set 2.** Statistical tests.

**Supplementary Data Set 3.** Microscopy settings for confocal imaging of reporter proteins.

**Supplementary Data Set 4.** Oligodeoxyribonucleotides used in this study.

**Supplementary Data Set 5.** Genome-wide poly-A RNA expression profile of Col-0, syy-1, syy-2 plants.

**Supplementary File 1.** Alignment of nuclear tDNA^Tyr^ introns.

**Supplementary File 2.** Machine-readable phylogenetic tree file.

## Dive Curated Terms

The following phenotypic, genotypic, and functional terms are of significance to the work described in this paper:

40S ribosomal protein gene Gramene: AT1G57540
40S ribosomal protein gene Araport: AT1G57540

Long non-coding RNA gene Gramene: AT1G08147
Long non-coding RNA gene Araport: AT1G08147

tRNA Tyr gene Gramene: AT5G61835
tRNA Tyr gene Araport: AT5G61835

tRNA Val gene Gramene: AT3G11395
tRNA Val gene Araport: AT3G11395

DECREASED DNA METHYLATION 1 (DDM1) Gramene: AT5G66750
DECREASED DNA METHYLATION 1 (DDM1) Araport: AT5G66750

NUCLEAR RNA POLYMERASE C, SUBUNIT 7 (NRPC7) Gramene: AT1G06790
NUCLEAR RNA POLYMERASE C, SUBUNIT 7 (NRPC7) Araport: AT1G06790

NUCLEAR RNA POLYMERASE D1A (NRPD1) Gramene: AT1G63020
NUCLEAR RNA POLYMERASE D1A (NRPD1) Araport: AT1G63020

NUCLEAR RNA POLYMERASE D1B (NRPE1) Gramene: AT2G40030
NUCLEAR RNA POLYMERASE D1B (NRPE1) Araport: AT2G40030

EXTENSIN22 (ETX22) Gramene: AT4G08380
EXTENSIN22 (ETX22) Araport: AT4G08380

EXTENSIN6 (EXT6) Gramene: AT2G24980
EXTENSIN6 (EXT6) Araport: AT2G24980

EXTENSIN9 (EXT9) Gramene: AT5G06630
EXTENSIN9 (EXT9) Araport: AT5G06630

EXTENSIN18 (EXT18) Gramene: AT1G26250
EXTENSIN18 (EXT18) Araport: AT1G26250

UBIQUITIN10 Gramene: AT4G05320
UBIQUITIN10 Araport: AT4G05320

CHROMOMETHYLASE 2 (CMT2) Gramene: AT4G19020
CHROMOMETHYLASE 2 (CMT2) Araport: AT4G19020

CHROMOMETHYLASE 3 (CMT3) Gramene: AT1G69770
CHROMOMETHYLASE 3 (CMT3) Araport: AT1G69770

DOMAINS REARRANGED METHYLTRANSFERASE 2 (DRM2) Gramene: AT5G14620
DOMAINS REARRANGED METHYLTRANSFERASE 2 (DRM2) Araport: AT5G14620

EXTENSIN1 (EXT1) Gramene: AT1G76930
EXTENSIN1 (EXT1) Araport: AT1G76930

EXTENSIN19 (EXT19) Gramene: AT1G26240
EXTENSIN19 (EXT19) Araport: AT1G26240

ARABINOXYLAN PECTIN ARABINOGALACTAN PROTEIN 1 (APAP1) Gramene: AT3G45230
ARABINOXYLAN PECTIN ARABINOGALACTAN PROTEIN 1 (APAP1) Araport: AT3G45230

SEED STORAGE ALBUMIN 3 (2S3) Gramene: AT4G27160
SEED STORAGE ALBUMIN 3 (2S3) Araport: AT4G27160
