# Supplementary material for: Reactivation of the tRNASer/tRNATyr gene cluster in Arabidopsis thaliana root tips
Source: Plant Cell. 2025 Jun 6;37(7):koaf137. doi: 10.1093/plcell/koaf137 (PMC12308677; doi:10.1093/plcell/koaf137)
Supplement: koaf137_Supplementary_Data [file koaf137_supplementary_data.zip › Supplementary_Figures_TPC_V13.pdf]

Supplementary Figure S1, Sequence and secondary structure of putative mature nuclear tRNA^Tyr transcripts

A

```
             *  *    *     *    *  *   *   *     ***     * ** **  ***
D-MAJOR   CCGACCUUAGCUCAGUUGGUAGAGCGGAAGACUGUAGAUCUUUAGGUCGCUGGUUCGAUUCCGGCAGGUCGGA
D-MINOR1  CCGACCUUAGCUCAGUUGGUAGAGCGGAAGACUGUAAAUCUUUAGGUCGCUGGUUCGAUUCCGGCAGGUUGAA
D-MINOR2  CCGACCUUAGCUCAGUUGGUAGAGCGGAAGACUGUAAAUCUUUAGGUCGCUGGUUCGAUUCCGGCAGGUCGGA
C-MAJOR   CCGACCUUAGCUCAGUUGGUAGAGCGGAGGACUGUAGAUCCUUAGGUCACUGGUUCGAAUCCGGUAGGUCGGA
C-MINOR1  CCGACCUUAGCUCAGUUGGUAGAGCGGAGGACUGUAGAUCCUUAGGUCAUUGGUUCGAAUCCGCUAGGUCGGA
C-MINOR2  CCGACCUUAGCUCAGUUGGUAGAGCGGAGGACUGUAGAUCCUUAGGUCAUUGGUUCGAAUCCGGUAGGUCGGA
C-MINOR3  CCGACUUUAGCUCAGUUGGUAGAGCGGAGGACAGUAGAUCCUUAGGUCACUGGUUCGAAUCCGGUAGGUCGGA
C-MINOR4  CCGACCUUAGCUCAGUUGGUAGAGCGGAGGACUGUAGAUCCUUAGGUCACUGGUUCGAAUUCGGUAGGUCGGA
C-MINOR5  CCGACCUUAGCUCAGUUGGUAGAGCGGAGGACAGUAGAUCCUUAGGUCACAGGUUCGAAUCCGGUAGGUCGGA
C-MINOR6  CCAACCUUAGCUCAGUUGGUAGAGCGGAGGACUGUAGAUCCUUAGGUCACUGGUUCGAAUUUGGUAGGUCGGA
C-MINOR7  CCGACCUUAGCUCUGUUGGUAUAGCGGAGGACUGUAGAUCCUUAGGUCACUGGUUCGAAUUCGGUAGGUCGGA
C-MINOR8  CCGACCUUAGCUCAGUUGGUAGAGCGGAGGACUGUAGAUCCUUAGGUCACUGGUUCGAAUCUGGUAGGUUUGA
C-MINOR9  CCGACCUUAGCUCAGUUGGUAGAGCGGAGGACAGUAGAUCCUUAGGUCAUAGGUUCGAAUCCGGUAGGUCGGA
```

B

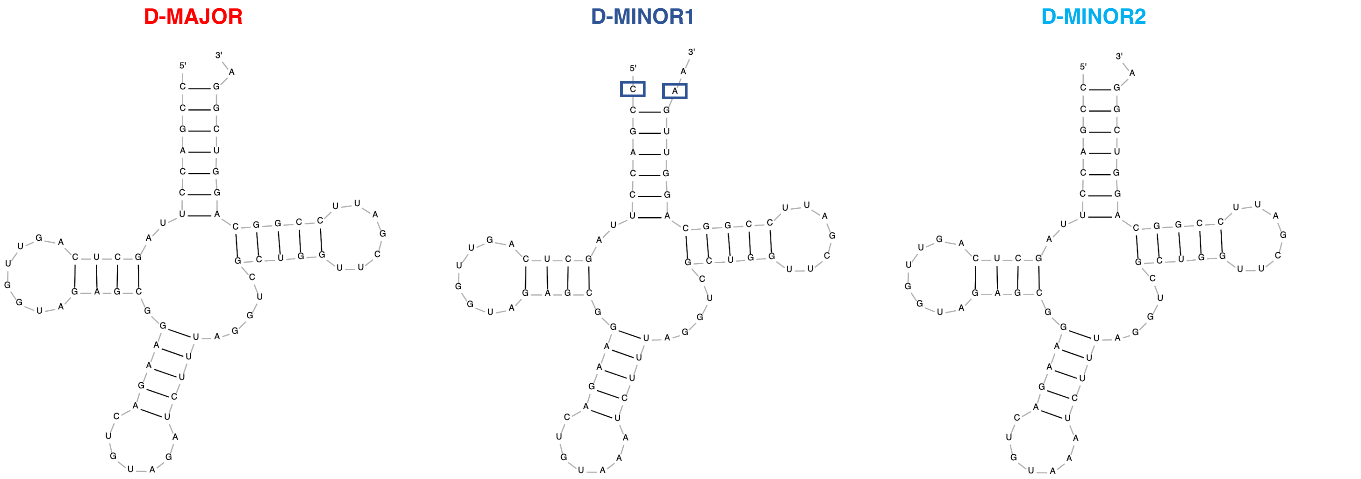

Supplementary Figure S1, Sequence and secondary structure of putative mature nuclear tRNA^{Tyr} transcripts

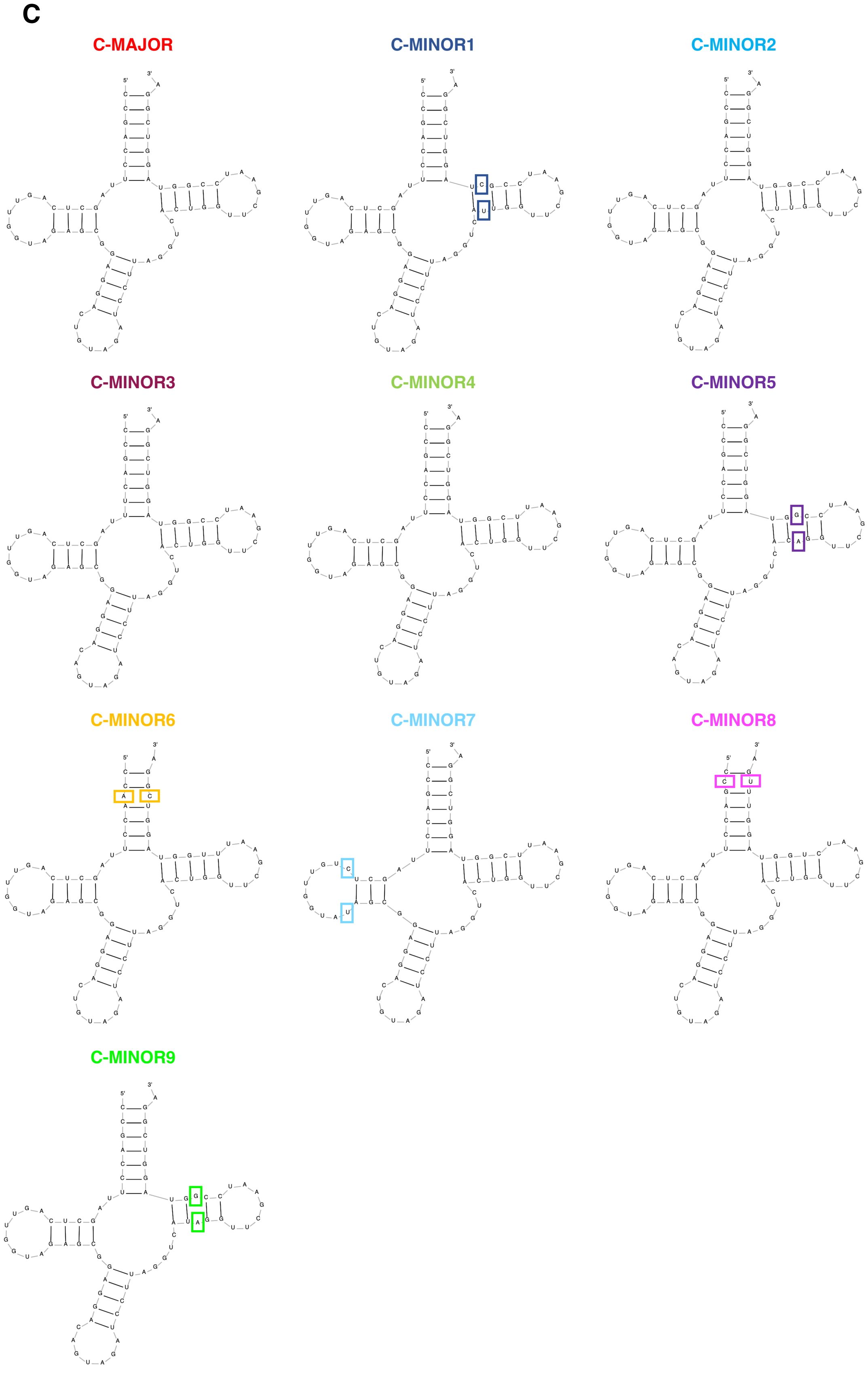

Supplementary Figure S1, Sequence and secondary structure of putative mature nuclear tRNA^{Tyr} transcripts

A, Alignment of mature tRNA^{Tyr} sequences, with polymorphic positions marked by asterisks. Underrepresented nucleotide polymorphisms (NPs) are highlighted in grey, and anticodons in yellow. The region recognized by D/C-Tyr probes is underlined. **B and C,** Secondary structures of dispersed and clustered mature tRNA^{Tyr} species, respectively. Mismatches in tRNA-like transcripts are indicated by squares. Supports Figure 1.

Supplementary Figure S2, Raw deletion zone sequences

> T3_*syy-1*_sequencing_result_forward

CAAACAAAGGATACGAAGAATCGTGATGTGACTGGGAGTTCCTTGGCACCCGAGAATTCCAGGTCCTGCCCTTGACACATACATATAACATA
TAGGTATTGGGCCTCTAATTTCGTGGCCCATTTTGTCTAAAATATTAATAGTGTCTTAAGAGTATCTTAAAAATAAATGAAAGAATTTAAGC
CCATTTTAGTAAAAATAAAATAGCATTTTGTTTCATTGTGAATTTTTTTTTTTTATAACCCACAATTAGCAAAATATAGAAAATTTAACAGT
TTAAAAAGCCTCCCCGAAAAATGACAAGACCGACACTGGCATCAAATTGATACATTTTCAGTTAACCGGCCATAACTCTTTAACCACAGCAC
CGGAGGATATTTAAACCGTTCGAGTTGTAGCCATCACTTTCGAATATCTACCAAATTTTTCCAAGAAGCATGTTTGCATATATTGTTGATAT
GCATTCTAATTTCTAACTTTGTTTACAATTTCTTAAAGAGGAATCACTCTGTATATTCTAAAGTACTGCAAAATCATTGTGTTCATATATTG
TTATAATTTTACACATGTATCAAATGCAATAGTACAGTTCGACCTTCGCTCAGTTGGAATTCTCGGGTGCCAAGGAACTCCAGTCACCGATG
TATCTCGTATGCCGTCTTCTGCTTGCCCTATAGTGAGTCCAAATAAAAAAA

> T7_*syy-1*_sequencing_result_reverse_complement

TTCACTAAAGGCAAGCAGAAGACGGCATACGAGATCGTGATGTGACTGGAGTTCCTTGGCACCCGAGAATTCCAGGTCCTGCCCTTGACACA
TACATATAACATATAGGTATTGGGCCTTTAATTTCGTGGCCCATTTTGTCTAAAATATTAATAGTGTCTTAAGAGTATCTTAAAAATAAATG
AAAGAATTTAAGCCCATTTTAGTAAAAATAAAATAGCATTTTGTTTCATTGTGAATTTTTTTTTTTTATAACCCACAATTAGCAAAATATAG
AAAATTTAACAGTTTAAAAAGCCTCCCCGAAAAATGACAAGACCGACACTGGCATCAAATTGATACATTTTCAGTTAACCGGCCATAACTCT
TTAACCACAGCACCGGAGGATATTTAAACCGTTCGAGTTGTAGCCATCACTTTCGAATATCTACCAGATTTTTCCAAGAAGCATGTTTGCAT
ATATTGTTGATATGCATTCTAATTTCTAACTTTGTTTACAATTTCTTAAAGAGGAATCACTCTGTATATTCTAAAGTACTGCAAAATCATTG
TGTTCATATATTGTTATAATTTTACACATGTATCAAATGCAATAGTACAGTTCGACCTTCGCTCAGTTGGAATTCTCGGGTGCCCAAGGAAC
TCCCAGTCACCCGATTATCTCGTAGCTTTTCGGC

> T3_*syy-2*_sequencing_result_forward

CACCAAAGGAACGAAGAATCGTGATGTGACTGGGAGTTCCTTGGCACCCGAGAATTCCAGGTCCTGCCCTTGACACATACATATAACATATA
GGTATTGGGCCTCTAATTTCGTGGCCCATTTTGTCTAAAATATTAATAGTGTCTTAAGAGTATCTTAAAAATAAATGAAAGAATTTAAGCCC
ATTTTAGTAAAAATAAAATAGCATTTTGTTTCATTGTGAATTTTTTTTTTTTATAACCCACAATTAGCAAAATATAAAAAATTTAACAGTTT
AAAAAGCCTCCCCGAAAAATGACAAGACCGACACTGGCATCAAATTGATACATTTTCAGTTAACCGGCCATAACTCTTTAACCACAGCTCGG
ATTAACCCACAATGACCTACAAAAACATACAAAACACACGAAAAGACACACAAAAACACTATAGAACTTCTAAAATGGAAAAAAACTCACTT
GGACTCTAAATAGACAAAACATAAGGAAAATACCAATAAAATACGATAAAAAATGATATTAAAAACCGTGATAAAGCGGTAAATATAGAGTA
TATCAGCAAGTTCGACACATGCCACTATGCCAGCTTACTAAAATATGGCGTCAAGGGTTTGTACAAGACTAATAAGCACGTCAATGCCTGAT
AGCTCGTAAGATATAGAAAGTAAAACACCTACAAATGCCTTTTCGTAAGAAGGTGTAAGAAATCTAGTGGAAACACTAAAAGAGGCAACCCT
GGTTCTCGGAAAACAGACGCAAGATCCTTCATGTTTGTTTATCGTGACCAAGATGTATTTTGAAGATTTCCAATCAGGCCCGGCCCAAGTGG
GTAGGGTAGAGGTGCTTAGGTTTAAAGCTCATCGAGTTGTAGCCATCACTTTCGAATATCTACCAGATTTTTCCAAGAAGCATGTTTGCATA
TATTGTTGATATGCATTCTAATTTCTAACTTTGTTTACAATTTCTTAAAGAGGAATCACTCTGTATATTCTAAAGTACTGCAAAATCATTGT
GTTCATATATTGTTATAATTTTACACATGTATCAAATGCAATAGTACAGTTCGACCTTCGCTCAGTTGGTAGACTAGGAGGTTGTTTCTTTG
CGCCAAGAACTGTGCAAAAACCACATGTGGGAGGGATCTTTTGGGTTTTGGAAGCCGGCCTAGTCAAGGGTTCAAACATCAAAAGGGGGCAA
GTAAAGGGAAAGTAACCTCTGAAATTTCCCCCCATTCCCTATAAAAAATTGGGAGGGAAGGGACTTTACCTTTCCAAAAAAAAAACTTGGAT
TTGGTTTTCCTTTTGGTTCCTTTTTAAAAAAATTTTTGGAAACCTTTGCAAAAAACCGAAAAGGGAAGAAAAACATTTTTAAAAAAAAAAAT
CACCCCCCCCGGATAAAAAATTTTCCCCAGGGGTGGGGTTTCCTTATGTTTTTTTTAAAAAAAAAAAAAACCCTTC

> T7_*syy-2*_sequencing_result_reverse_complement

TTTTTTATAACCCAAAAAAGGCAGCCCCTCTATTTATTTTTTTTTTTTGCCCCGCCTTTTTTTCCCCGGGTTGGTTCCCCCTAGTTTTTAAAA
AAATTAACACCCGAAAACCCCAAAAACCCTTTATTTTTTTTAAAAAAGAAAAACATCCCCCTTGTGTCTCTAAAACACAAAACCTGGGGAAA
CCCCCCAAAAATGGGTAAAAAATTGTTTTAAACCCGGGAAAAAGGCGGTAATTTGAGGTTAATCCCCATTTTCGCCCCCTCGCCCCTTTTCC
CCGCTTTTTTAAAAGGGGTCAAGGGTTTTTACAAAATTAAAAAGCCCGTCAATCCCTGATAGTTTGTAAGATTAGAAAGTAAAACCCCTACA
AATGCCTTTTTGTAAGAAGGTGTAAGAAATCTAGTGGAAACACTAAAAGAGGCAACCCTGGTTCTCGGAAAACAGACGCAAGATCCTTCATG
TTTGTTTATCGTGACCAAGATGTATTTTGAAGATTTCCAATCAGGCCCGGCCCAAGTGGGTAGGGTAGAGGTGCTTAGGTTTAAAGCTCATC
GAGTTGTAGCCATCACTTTCGAATATCTACCAGATTTTTCCAAGAAGCATGTTTGCATATATTGTTGATATGCATTCTAATTTCTAACTTTG
TTTACAATTTCTTAAAGAGGAATCACTCTGTATATTCTAAAGTACTGCAAAATCATTGTGTTCATATATTGTTATAATTTTACACATGTATC
AAATGCAATAGTACAGTTCGACCTTCGCTCAGTTGGTAGACTAGGAGGTTGTTTCTTTGCGCCAAGAACTGTGCAAAAACCACATGTGGATG
GATCTTTTGGTTTTGTAGTCTGCCTAGTCAATGGTTCAAACATCATAAGGTGTCAAGTATAGTGAAAGTAACCTCTGACATTTCCACTCATT
CCCTATAACAAACTTGGCATGTAACGTAACTTAACCTTTTACCAAAAAAAAAAAACTTGACATGTATGCATTTCTCTTATTGAGTTCTCTTT
TGATAACGATTTTGTTGTAATACTCTTGTCAAAGAACACTGAAAATGGAATGAAACAAGCTTTAGAAAATAAGATCTGACCCTACCGGATTC
AAACTATTGACCTAAGGATGTTCTGCTTCAACTATTGTTTTCATTCGAAATGCAAAGAAACAACCTCCGTTCCAAAAAGCTAGATCTTTGGC
CTCCATAGCTTGCAGAATTCACTTGATTAAGTTATGTTCTCCATGACATTATAATACCACAGGCTTATATGCTGTGTCTATATGACAATATC
ACTGAAATCCTAGCTCTTTACTCACATTCAAAACACATTGATGTCGATTATAATTAATTAATTAATTATGCTCGAGAAATGGTAGCATTAGG
TTTGTTGGTGATCAAACATATTCCTACACATATTTAACTAGCAGGCATCTCCATGAAGTCACAACCTCGGTGTGGTGGAATTCTCGGGTGCC
AAGGAACTCCCAGTCACCCGATGTATCTTCGTTGCTTTCTGT

Supplementary Figure S2, Raw deletion zone sequences

The color code follows that of Fig. 3A. Green and yellow regions indicate genomic sequences upstream and downstream of the SYY cluster deletion, amplified with 3 + 2 tailed primers (red). GC/TC dinucleotides highlighted in grey in the repair zone are shared by both ends, thus their origin cannot be determined. Supports Figure 3E.

Supplementary Figure S3, Phenotyping of tRNA gene mutants during development

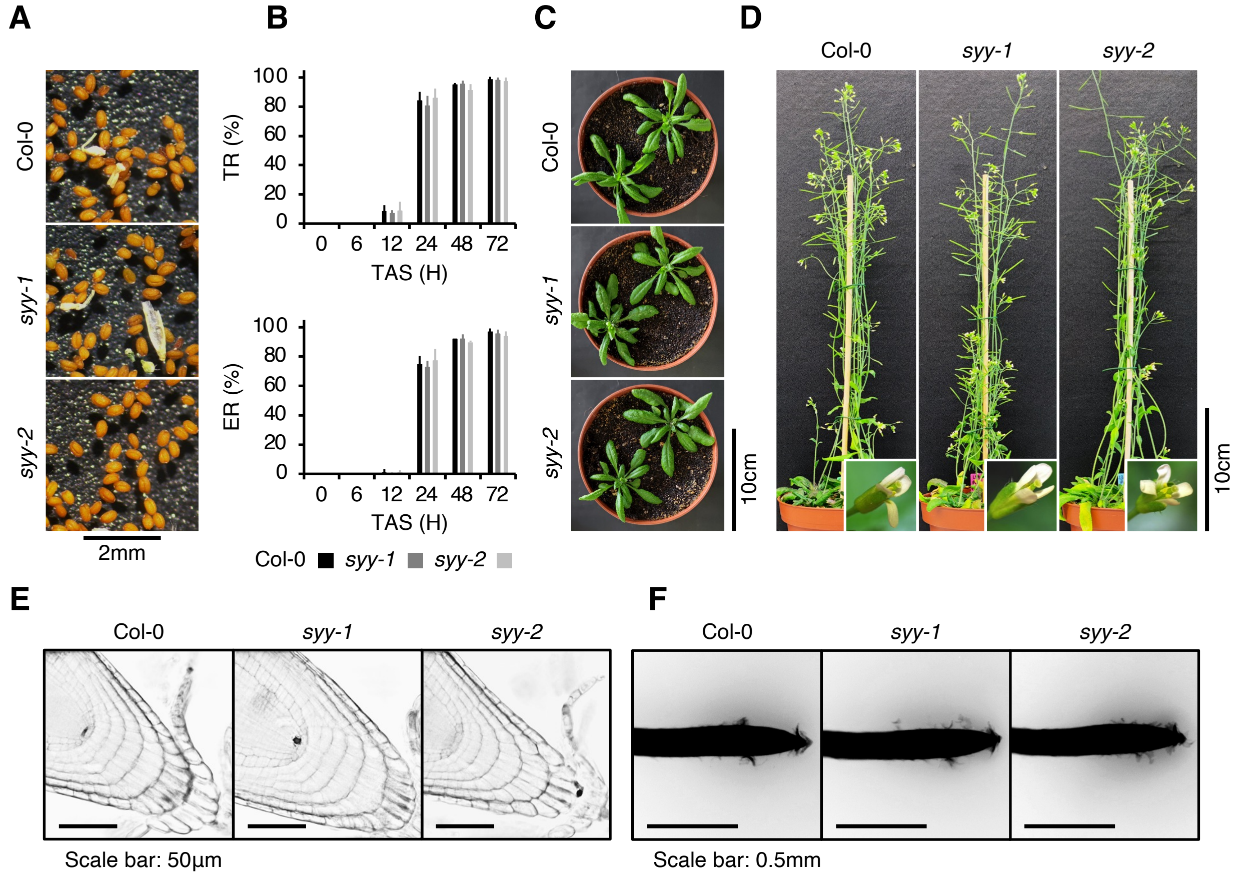

Supplementary Figure S3, Phenotyping of tRNA gene mutants during development

A, Dry seeds of Col-0, *syy-1*, and *syy-2*. **B,** Testa and endosperm rupture kinetics (TR and ER, respectively) during germination of Col-0, *syy-1*, and *syy-2* seeds. Histograms represent the mean of three independent biological replicates with standard deviations. A biological replicate refers to the independent repetition of the experiment under identical conditions. A two-way ANOVA indicated no significant differences in rupture kinetics between Col-0, *syy-1*, and *syy-2* ($P = 0.603$ and 0.607, respectively). Please refer to **Dataset S2** for a full description of the statistics. TAS: time after stratification. **C and D,** Rosettes, flowers, and bolted plants with pods of Col-0, *syy-1*, and *syy-2*. **E,** Propidium iodide staining of 7DAS Col-0, *syy-1*, and *syy-2* root tips. **F,** Nigrosin staining of 7DAS Col-0, *syy-1*, and *syy-2* root tips. Supports Figures 3 and 4.

Supplementary Figure S4, Consensus sequence used for the mapping of NGS data

CNAGGGTNGTGAAATANCNTTTNNNACTNTGTTGCNNGNATTTGTTTGGCTTTATTTTCAAGAATATAAACAATNATNNGCNCANTCTCAATATTACATGCATAAATATANCATCAAAGT
TGCTCTTTAGAAATAATTTANTTTTTGANNTGAGAAAATNTTATAAAAATTNNAAANAATCAAGACAGCCAAATAGGTTTATGGTTCTNATNTTTTTTATTNTGTTCATAAACTNNNTNA
TCCTTGNAATNTTCTACTTCTNACTCATGATCAAAATTTAGGATANAGAATGGTATANNGTTTAGTCTATTTTGAAATAAAAGTTTAGTTTANAGAAAANGTGGANNTGNCGGAGTGNNG
TTATCGGGNATNACTAGAAATCATGNNGGNTTTGCCCGCGCANGTTNGAATCNTGCCGTTNACGTTTTTATTTTGAGTGTTGGTNAGTTNTTANAATNACTTANTGTGTTTTATGNATNG
TNTTNNAATTTGAACNGTTTTNCATCATGTTACNGTTTTCAGTAAANTTTAGTATTGATNAAAAAANNTNNNNAAAAAACAANANAGCATGAAAGATATGAAGTCAACTTTNTTTCTANT
CCTCTTCNNNANAAGTTTAAATTTGATTAAAAAGNTNNNNTNGAATCATCAACATGCNTAAAGTGTTATNATACNAAACCGACCTTAGCTCAGTTGGTAGAGCGGAGGACTGTAGTTGAN
GCAGATNATCCTTAGGTCACTGGTTCGAATCCGGTAGGTCGGATCATNAAANTTNAAANNNTTNTTTNNTNCATTTCGTTTCAAAGNGNTTAGANAAGAGTATAACTACAAACTNNTTTT
TCTATNANAANTTNGTTTNAGAGAATTGCATAGCTANTGANNGTATTATCANAAANGAGTGGGAANTCTNNAAGNAATTTTCACTGTANTTTAAACCGTTNAAGNNNNNNNNNNNNNNN
NNNNNNNTTACGAGNTTTANGCAANNNNNNNNGNNTTTTTCNAATATAATGTTTTNATAAATTCAAACTTTNTTTNCAAANTTNTAATANAGANTCACTATGANTATGCTAACTTAATAC
AAATCATTNTGNTNATAGAATATTTNGATCAGTACACATGCATGAAATANAATACAATCCGACCTTAGCTCAGTTGGTAGAGCGGAGGACTGTAGNAGACGNAGATTATCCTTAGGTCAC
TGGTTCGAATCCGGTAGGTCGGAATTTGCTCCCACANGAGANCTTTTTATTTTTCTTTNGNTGTGACATTAANNTNTTTNNAATTTTATAATAAACGGTTATATGGTGGTCGACAATTNA
ACATACCNAAAAGTTTNGCTCGNATTNTTNAANGATCCGTATGTTTANCNTTTTTCAATATTGATACGATGANAAATGATTTAAAAGTGANTAAAAACTATGAGTTTTCTAATTTNCTTT
GGTCAAACAAANATTTAGTTTTANAGTTTTAAAAANNACAAANAATGTNTGCATANTTATNTTCAAATGCTTGTGGGTTGTGCCTATAAGTTGTCAACGTTCATA

Supplementary Figure S4, Consensus sequence used for the mapping of NGS data

The color code follows that in Figure 3A: C-tDNA^{Ser} is green, C-tDNAs^{Tyr} are blue, and introns are gray. Positions with multiple bases in the SYY repeat alignment are marked as degenerate (red Ns). Y1 and Y2 positions were blocked using the C-MAJOR sequence for targeted read mapping. Supports Figure 6E.

Supplementary Figure S5, Design of a reporter system to monitor Ser, Tyr, and Pro-rich translation

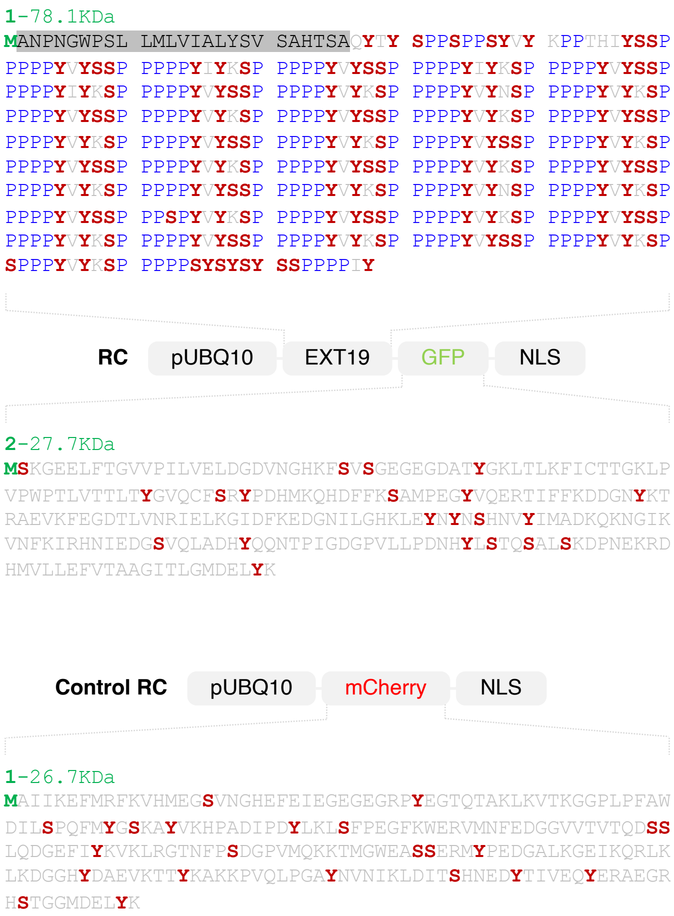

Supplementary Figure S5, Design of a reporter system to monitor Ser, Tyr, and Pro-rich translation

In protein sequences, initiator Met residues are green, Ser and Tyr are red, and Pro stretches are blue. The predicted molecular weight of translational products is indicated in green. The removed EXT19 secretion motif is shaded in gray. Supports Figure 9A.

Supplementary Figure S6, Uncropped blots

Figure 2B, Roti staining

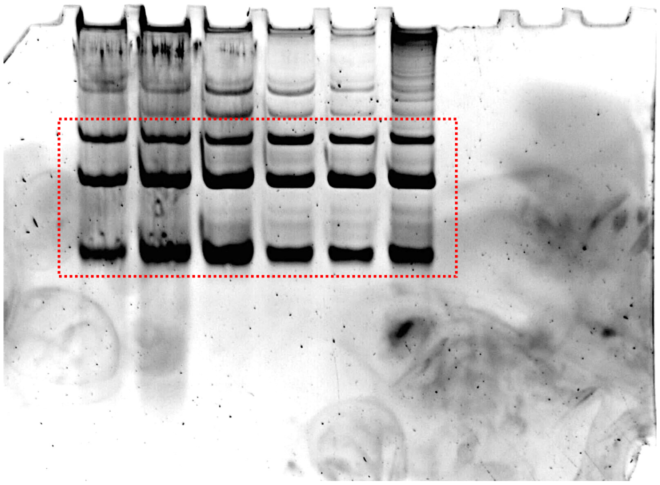

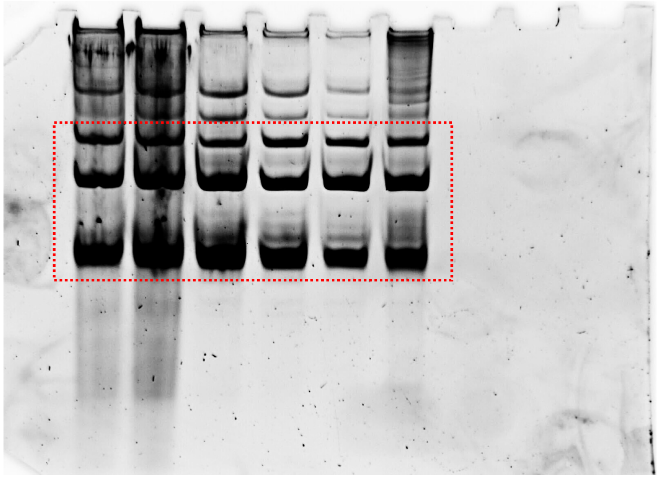

Figure 2B, D/C-Tyr E1

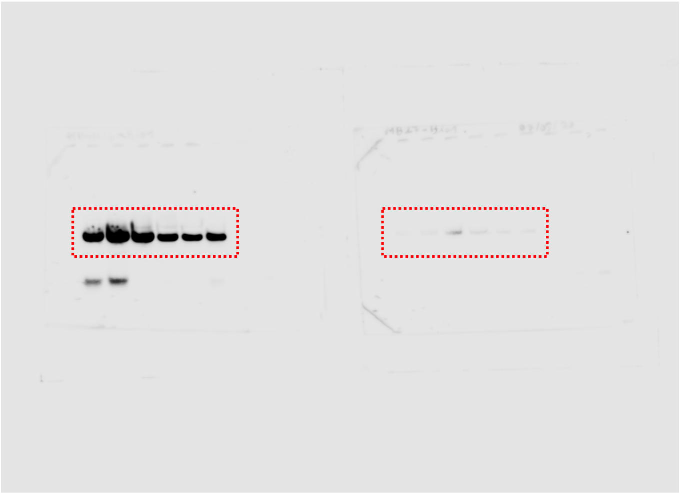

Figure 2B, D/C-Tyr E2

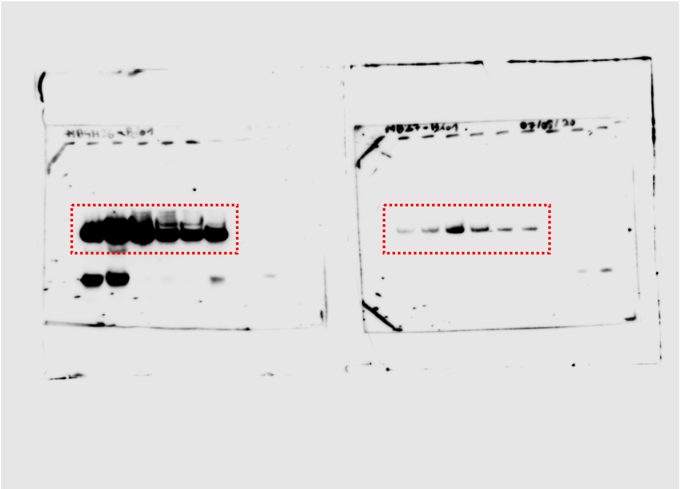

Figure 2B, D-Ala

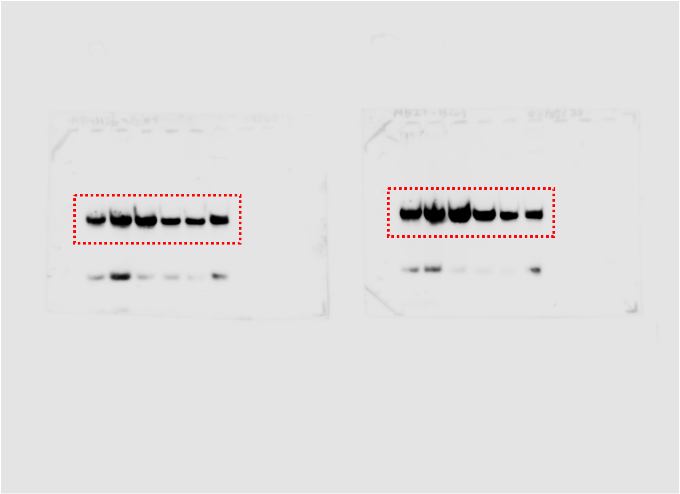

Supplementary Figure S6, Uncropped blots

Figure 2D, Roti staining

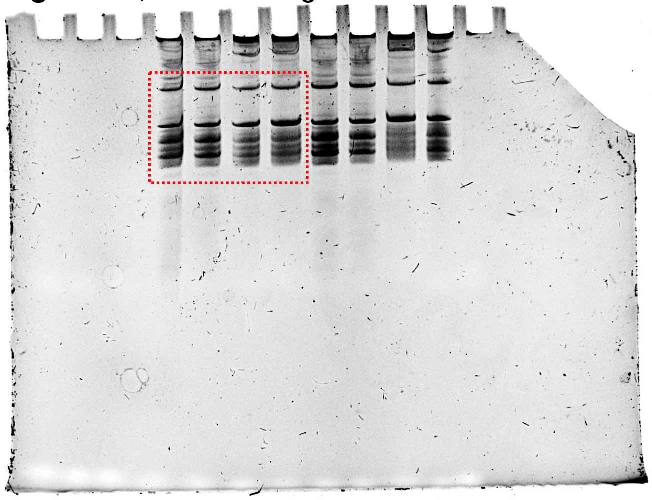

Figure 2D, C-Tyr

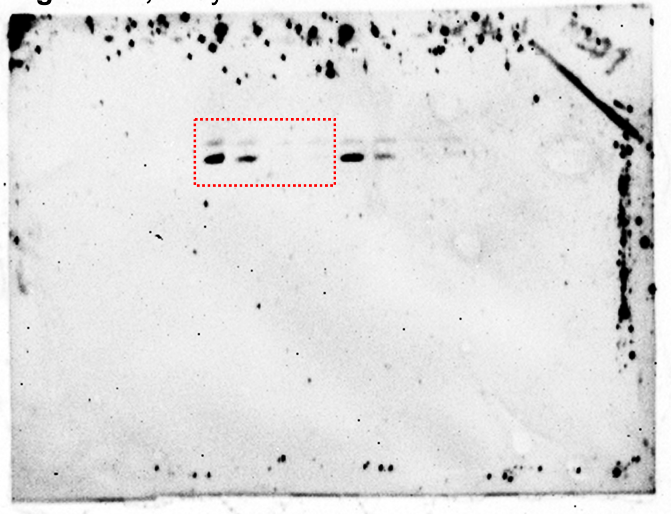

Figure 2D, D-Ala

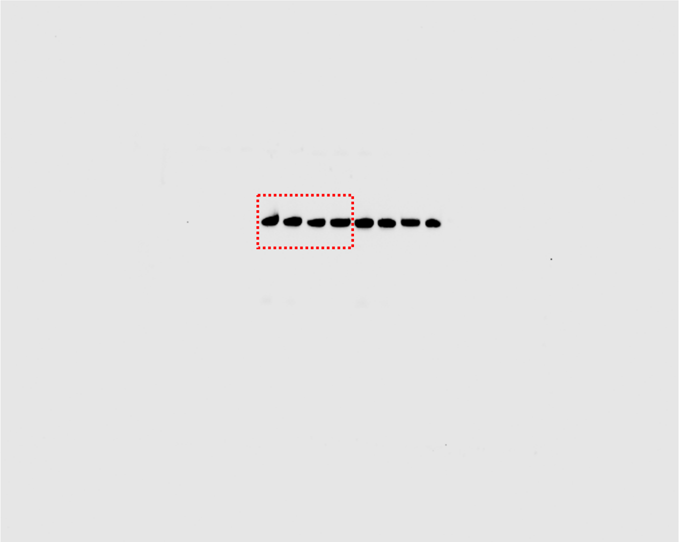

Supplementary Figure S6, Uncropped blots

Figure 3C, Roti staining

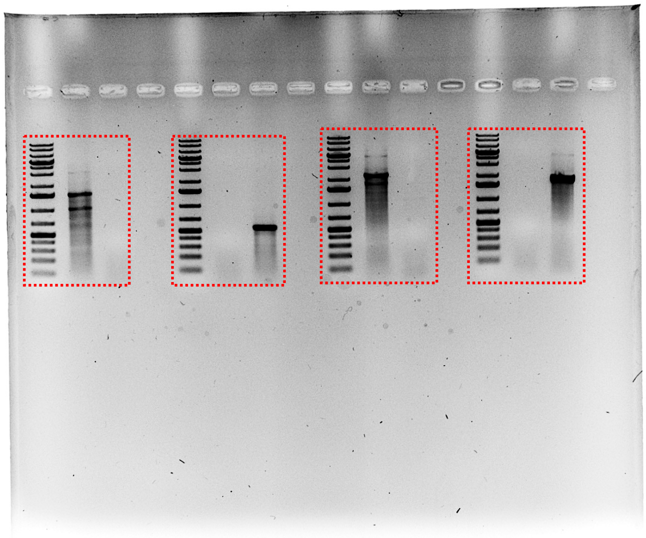

Figure 3D, Roti staining

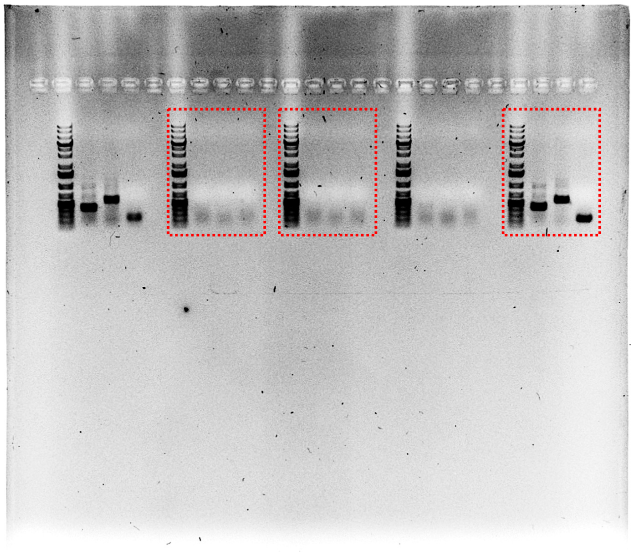

Supplementary Figure S6, Uncropped blots

Figure 3G, Roti staining

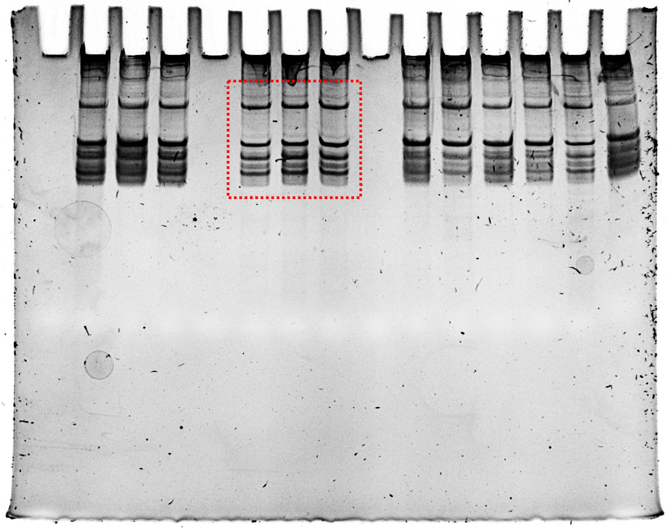

Figure 3G, D-Tyr

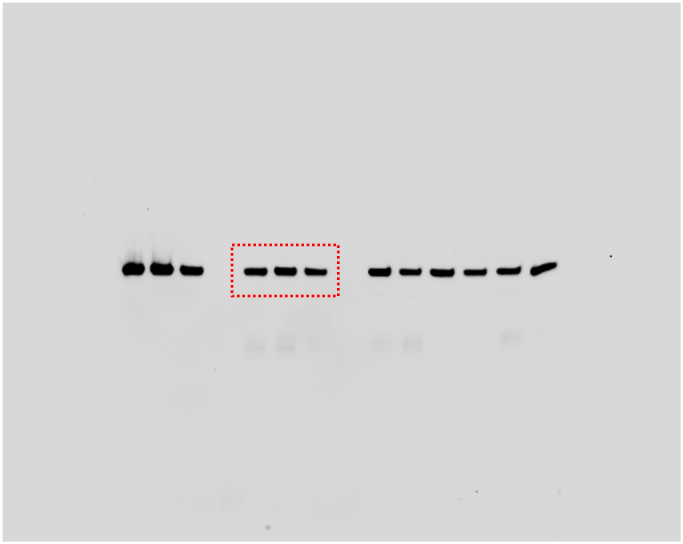

Figure 3G, C-Tyr

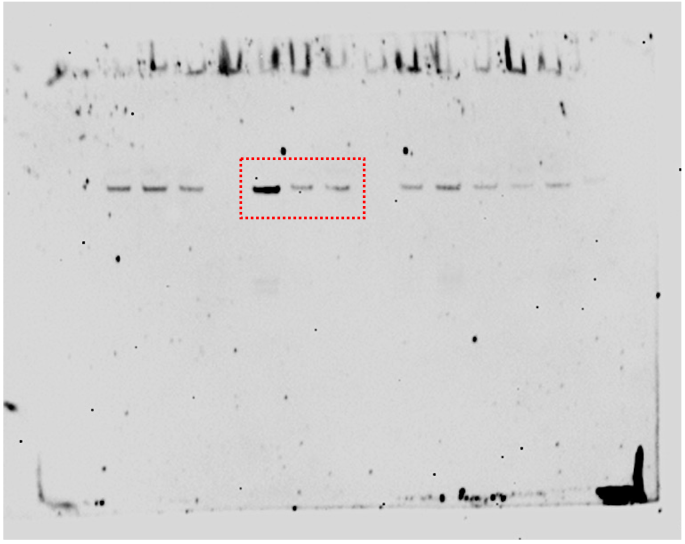

Figure 3G, D-Ala

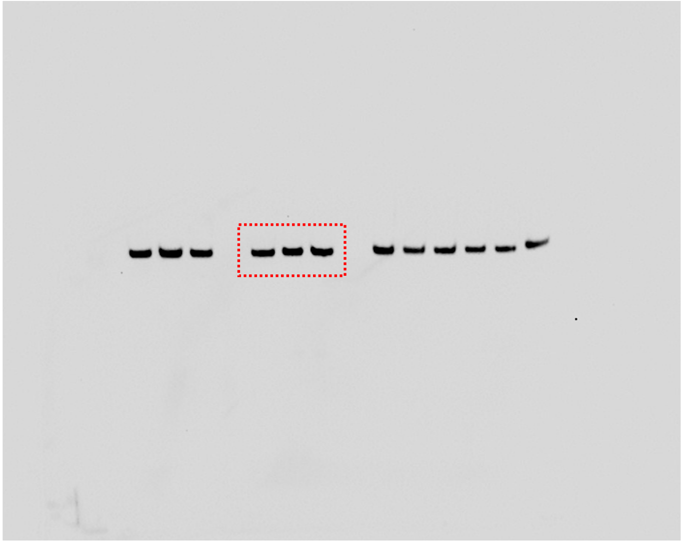

Supplementary Figure S6, Uncropped blots

Figure 5D, p probe

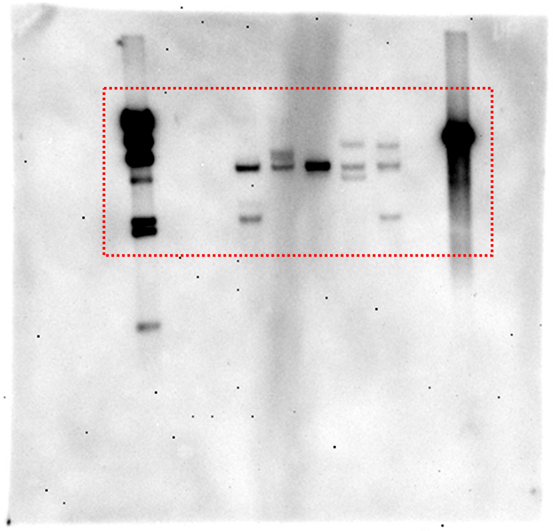

Supplementary Figure S6, Uncropped blots

Figure 5E, Roti staining

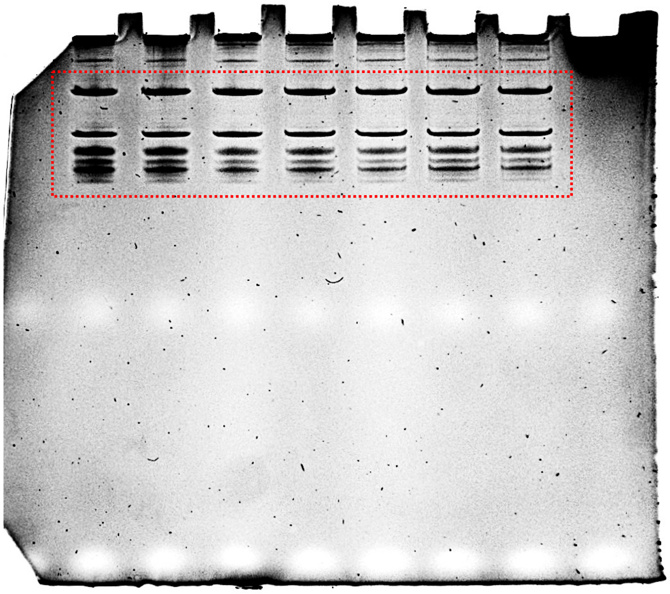

Figure 5E, C-Tyr

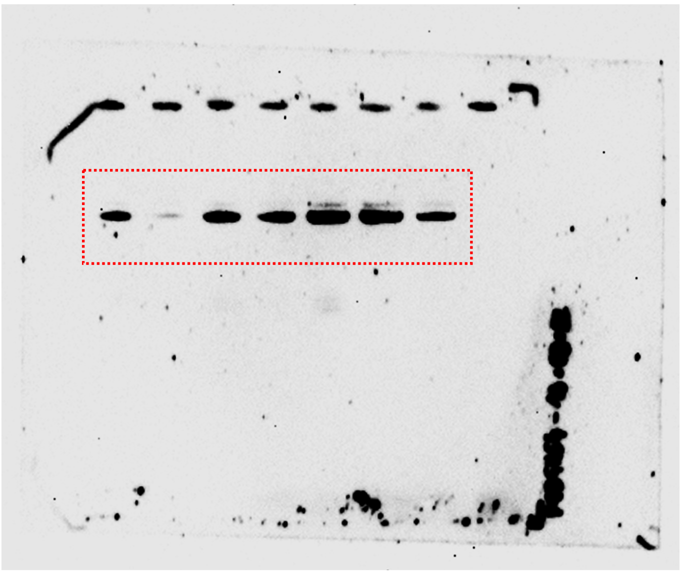

Figure 5E, D-Ala

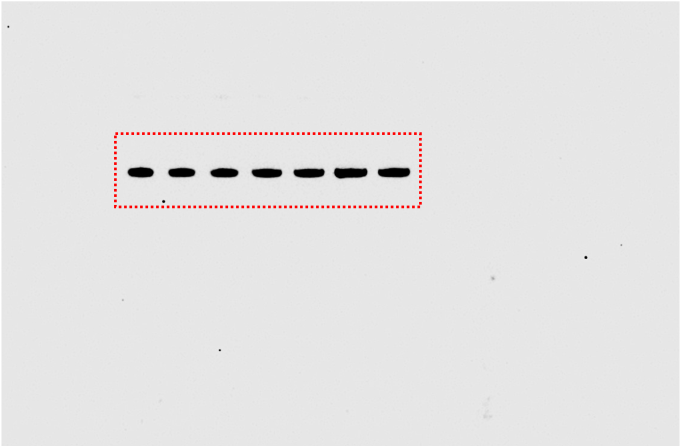

Supplementary Figure S6, Uncropped blots

Figure 5G, Roti staining

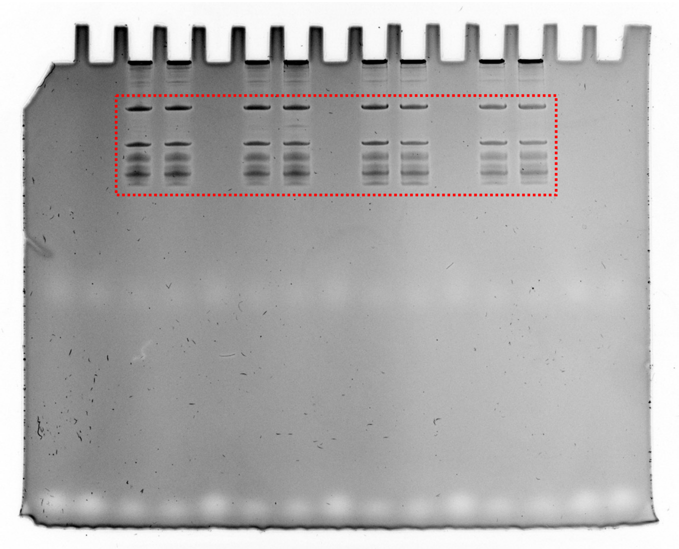

Figure 5G, C-Tyr

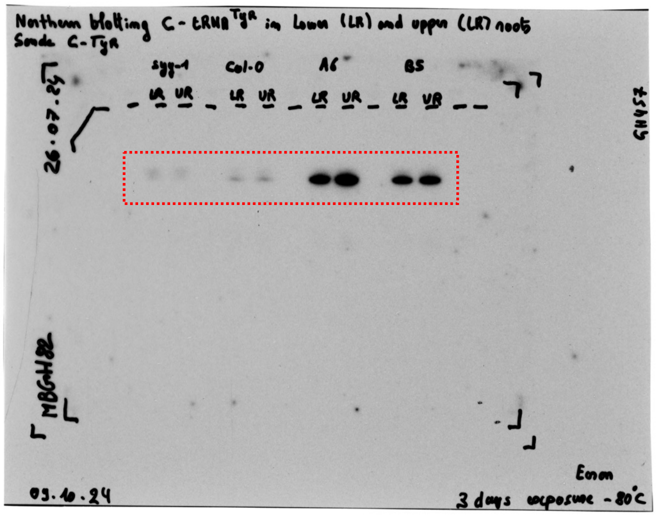

Figure 5G, D-Ala

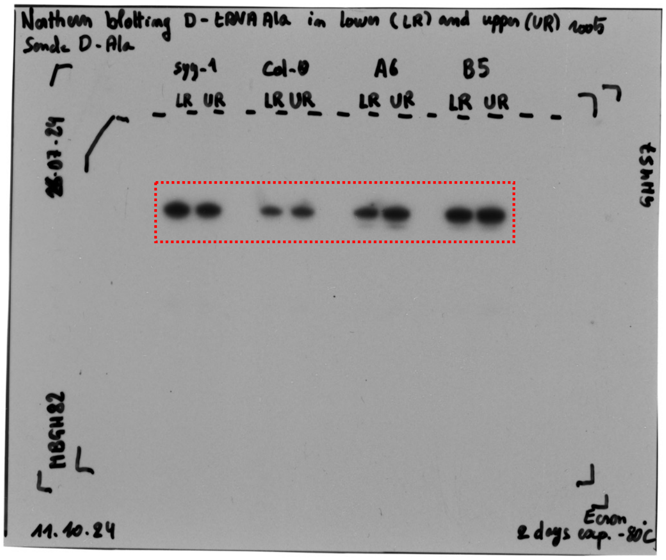

Supplementary Figure S6, Uncropped blots

Figure 6C, Roti staining Val PCR

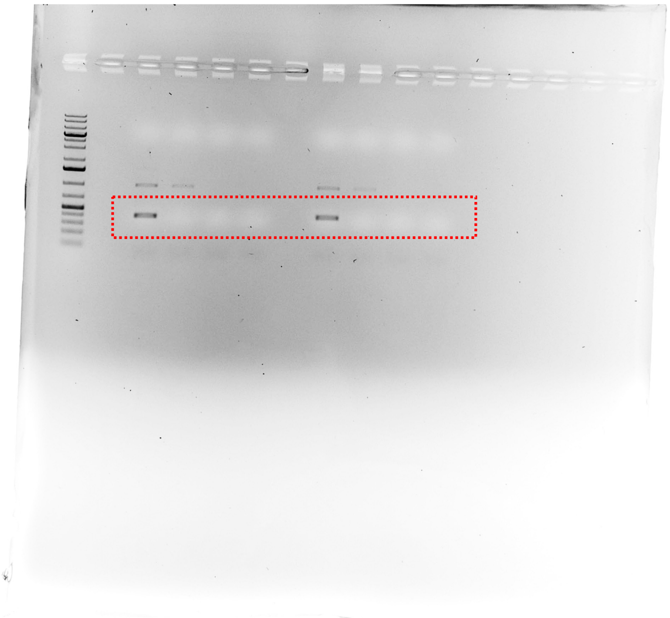

Figure 6C, Roti staining Y1 PCR

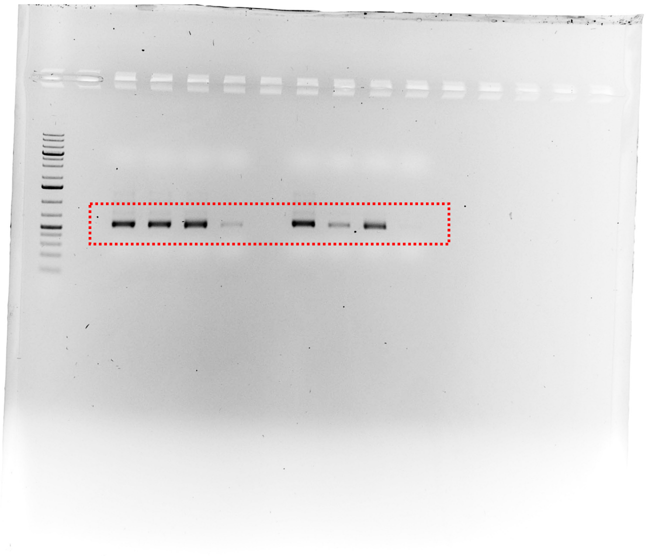

Figure 6C, Roti staining Y2 PCR

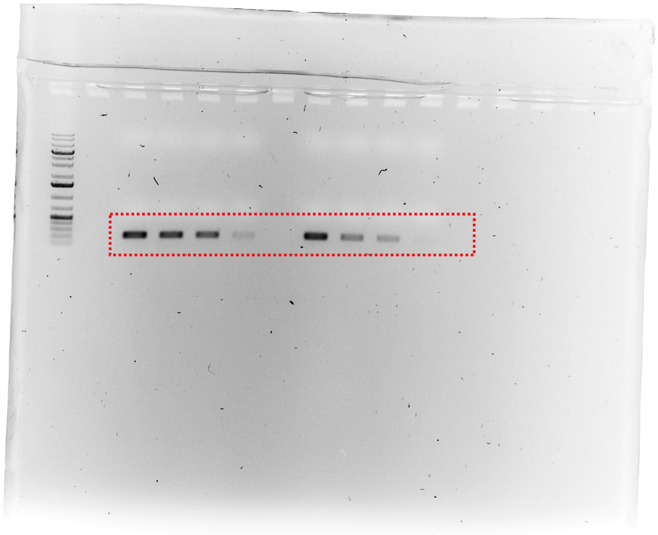

Supplementary Figure S6, Uncropped blots

Figure 6I, Roti

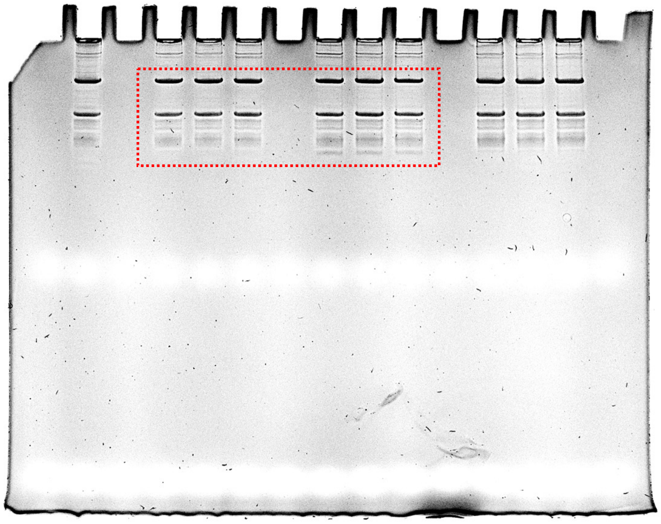

Figure 6I, C-Tyr

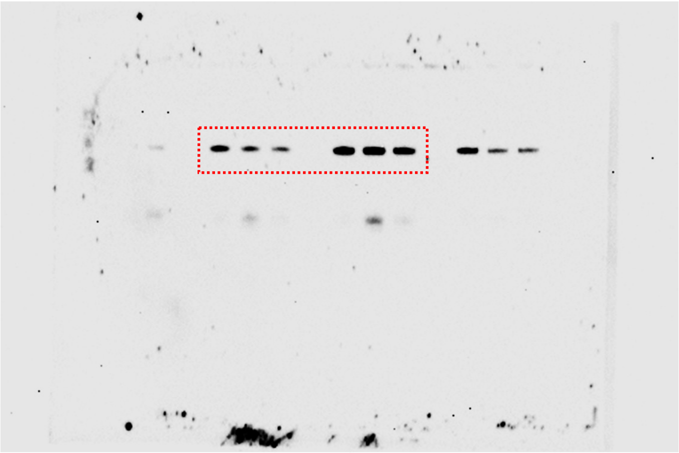

Figure 6I, D-Ala

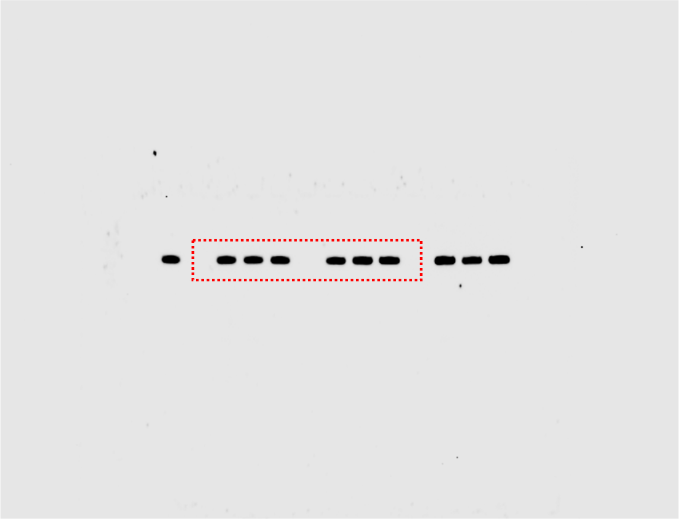

Supplementary Figure S6, Uncropped blots

Figure 7A, Roti staining Val PCR

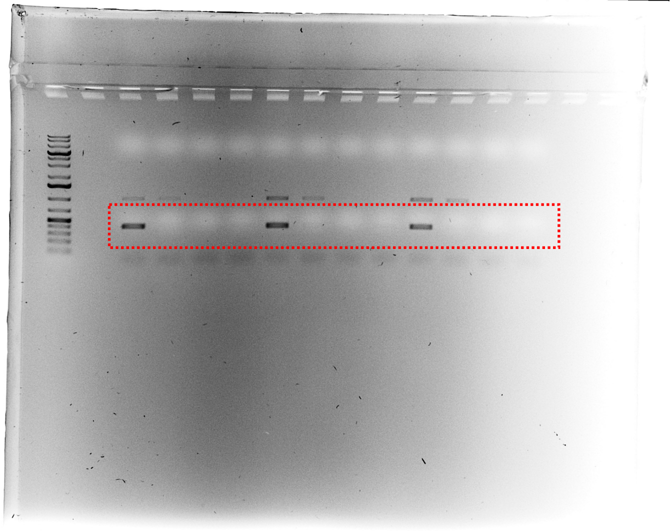

Figure 7A, Roti staining Y1 PCR

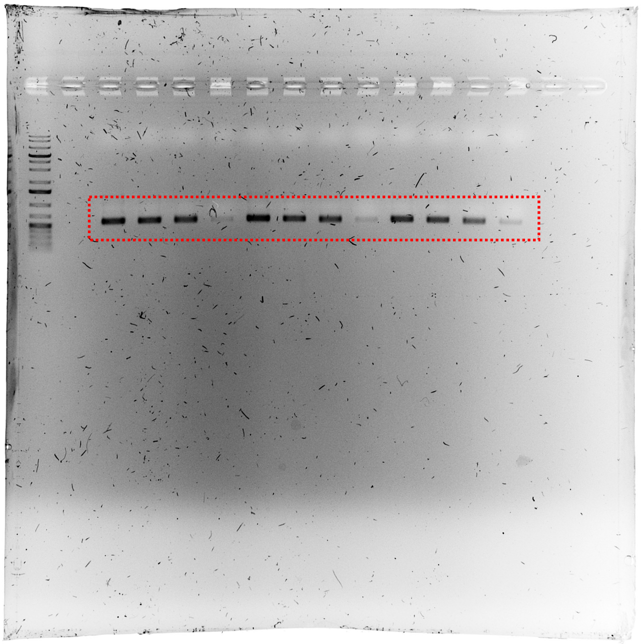

Figure 7A, Roti staining Y2 PCR

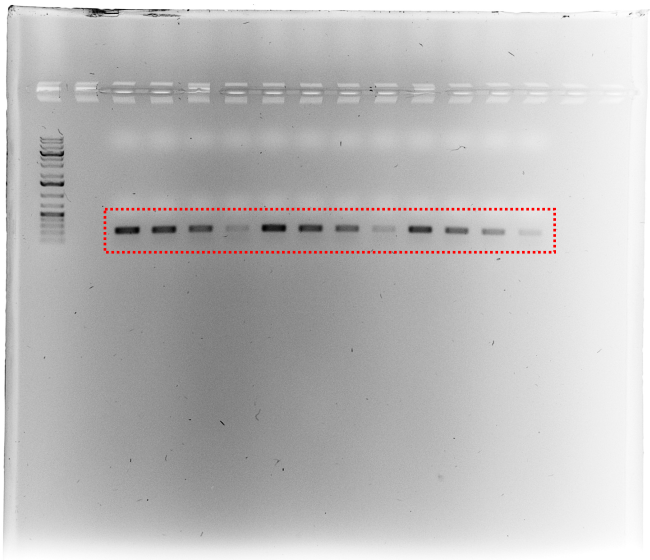

Supplementary Figure S6, Uncropped blots

Figure 7C, Roti staining Val PCR

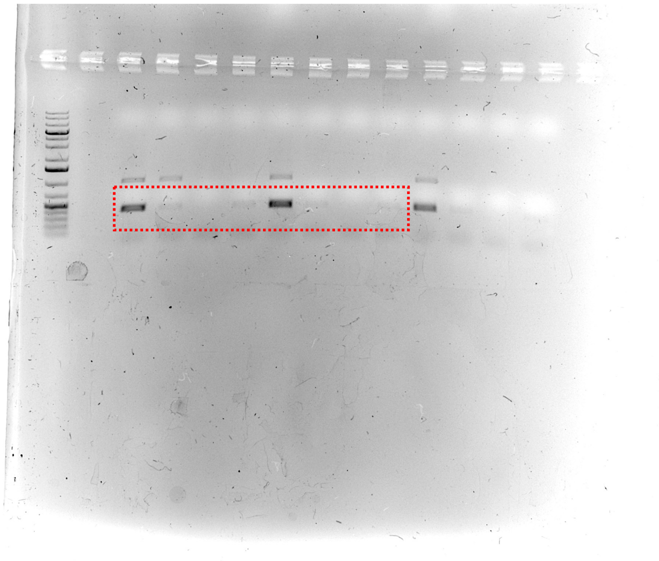

Figure 7C, Roti staining Y2 PCR

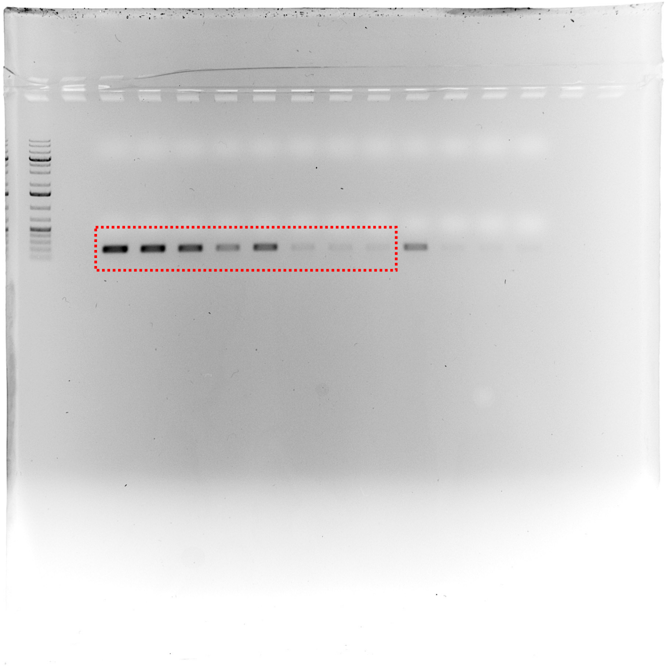

Supplementary Figure S6, Uncropped blots

Figure 7E, Roti staining

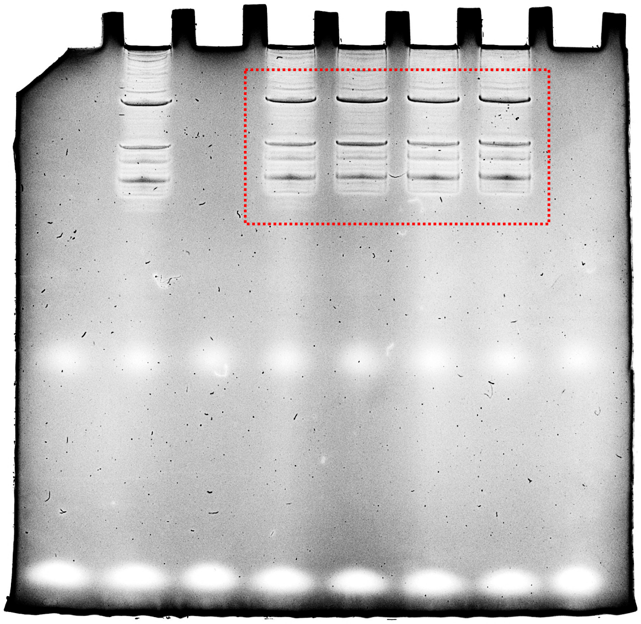

Figure 7E, C-Tyr

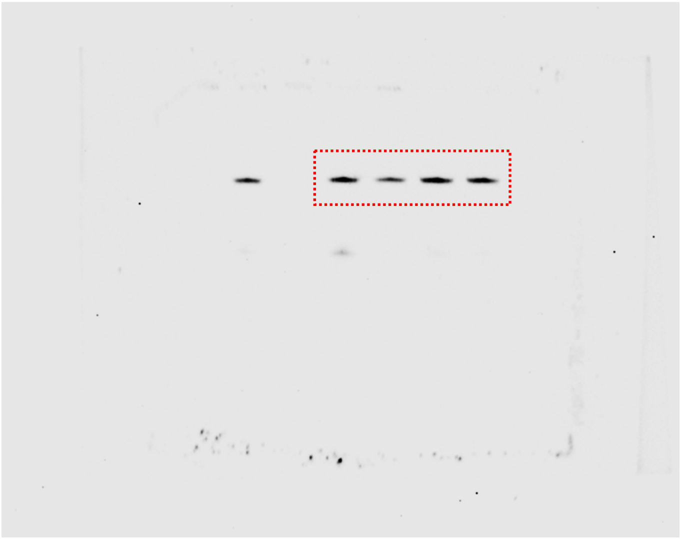

Figure 7E, D-Ala

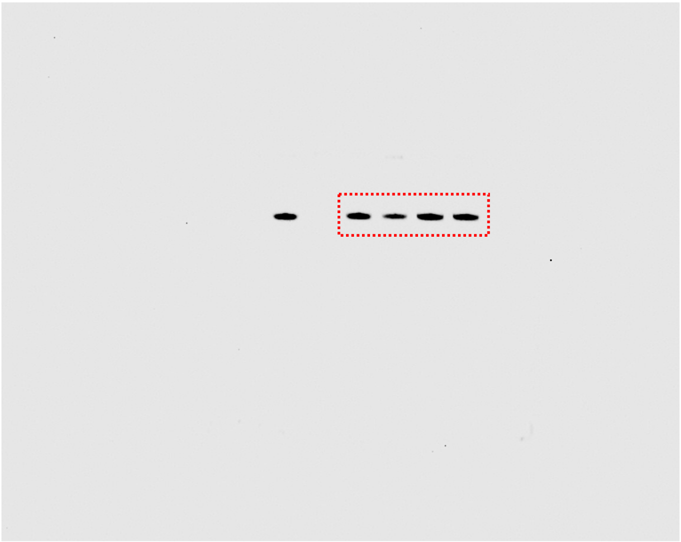

Supplementary Figure S6, Uncropped blots

Figure 10F, Coomassie staining

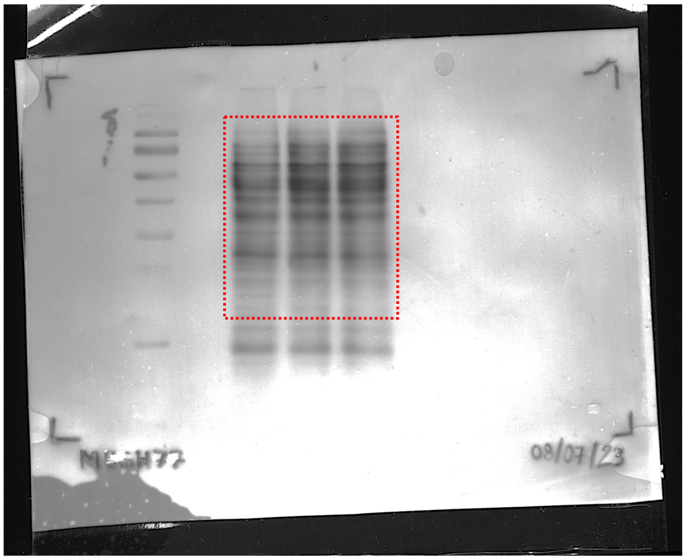

Figure 10G, Coomassie staining

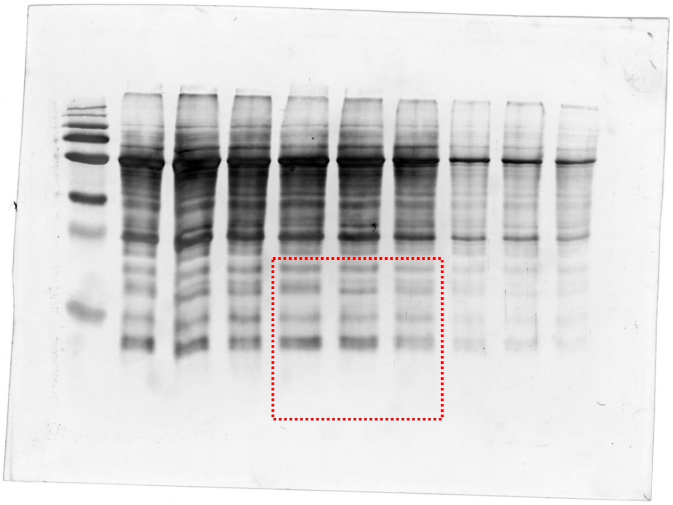

Figure 10F, Western blot @GFP

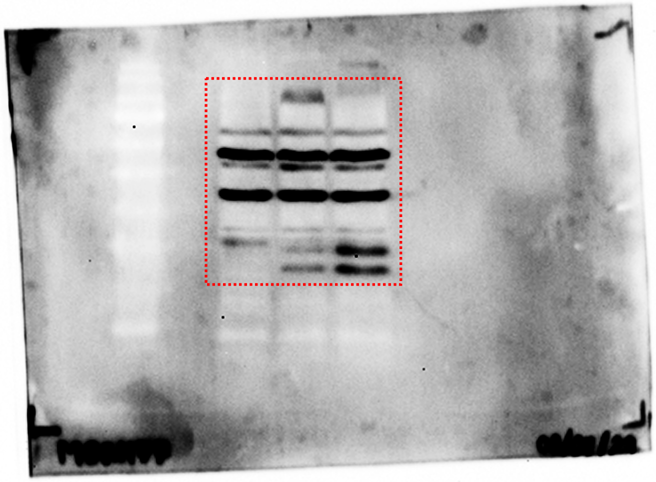

Figure 10G, Western blot @mCherry

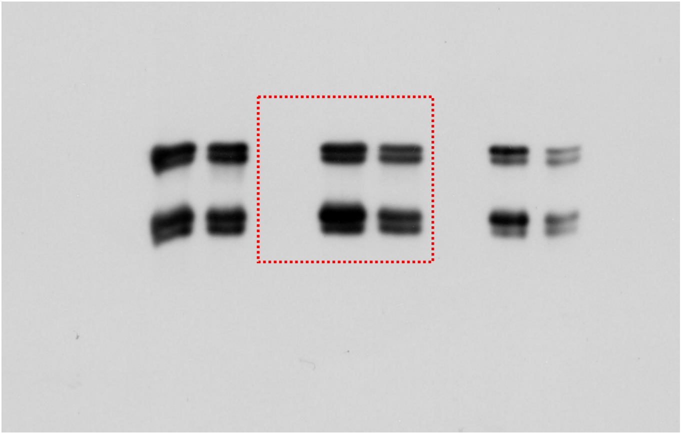

Supports Figures 2B, 2D, 3C, 3D, 3G, 5D, 5E, 5G, 6C, 6I, 7A, 7C, 7E, 10F, and 10G.
