# Supplementary material for: Reactivation of the tRNASer/tRNATyr gene cluster in Arabidopsis thaliana root tips
Source: Plant Cell. 2025 Jun 6;37(7):koaf137. doi: 10.1093/plcell/koaf137 (PMC12308677; doi:10.1093/plcell/koaf137)
Supplement: koaf137_Supplementary_Data [file koaf137_supplementary_data.zip › Supplementary_File_S1_V13.docx]

**Supplementary File S1, Alignment of nuclear tDNA^Tyr^ introns**

**> D-VAR1**

----TGTTTGCAGACA-

**> D-VAR12**

----TGTTTGCAGATA-

**> D-VAR8**

----GCGTTGCAGATA-

**> C-VAR1**

----TTGACGCAGATT-

**> C-VAR4**

----TTGACGCAGATA-

**> C-VAR6**

----TTGAAGCAGATA-

**> C-VAR2**

----TAGACGCAGATT-

**> C-VAR3**

----TAGACGTAGATT-

**> C-VAR5**

----AAGACGCAGATT-

**> D-VAR3**

TTGTTTAAAACAGATA-

**> D-VAR11**

TTGTTTGCAACAGAAA-

**> D-VAR2**

T---CTTTGACAGATA-

**> D-VAR9**

T---CTGTGACAGCAA-

**> C-VAR7**

----TTGACGC------

**> D-VAR4**

----TAGTTGCTGTG--

**> D-VAR5**

----TAGTTGCTGTA--

**> D-VAR6**

----TAGTAGCTGTA--

**> D-VAR10**

----TAGTTGCTGAA--

**> D-VAR7**

TGGGTAATTCCAAAGAA

Supports Figure 1B.
